# Tumor-Associated Macrophages in Human Breast, Colorectal, Lung, Ovarian and Prostate Cancers

**DOI:** 10.3389/fonc.2020.566511

**Published:** 2020-10-22

**Authors:** Irina Larionova, Gulnara Tuguzbaeva, Anastasia Ponomaryova, Marina Stakheyeva, Nadezhda Cherdyntseva, Valentin Pavlov, Evgeniy Choinzonov, Julia Kzhyshkowska

**Affiliations:** ^1^ Laboratory of Translational Cellular and Molecular Biomedicine, National Research Tomsk State University, Tomsk, Russia; ^2^ Cancer Research Institute, Tomsk National Research Medical Center, Russian Academy of Sciences, Tomsk, Russia; ^3^ Department of Pathophysiology, Bashkir State Medical University, Ufa, Russia; ^4^ Department of Urology, Bashkir State Medical University, Ufa, Russia; ^5^ Institute of Transfusion Medicine and Immunology, Medical Faculty Mannheim, University of Heidelberg, Mannheim, Germany; ^6^ German Red Cross Blood Service Baden-Württemberg—Hessen, Mannheim, Germany

**Keywords:** tumor-associated macrophage, monocyte, CD68, lymphatic metastasis, hematogenous metastasis, chemotherapy, immunotherapy, biomarker

## Abstract

Tumor-associated macrophages (TAMs) are major innate immune cells that constitute up to 50% of the cell mass of human tumors. TAMs are highly heterogeneous cells that originate from resident tissue-specific macrophages and from newly recruited monocytes. TAMs’ variability strongly depends on cancer type, stage, and intratumor heterogeneity. Majority of TAMs are programmed by tumor microenvironment to support primary tumor growth and metastatic spread. However, TAMs can also restrict tumor growth and metastasis. In this review, we summarized the knowledge about the role of TAMs in tumor growth, metastasis and in the response to cancer therapy in patients with five aggressive types of cancer: breast, colorectal, lung, ovarian, and prostate cancers that are frequently metastasize into distant organs resulting in high mortality of the patients. Two major TAM parameters are applied for the evaluation of TAM correlation with the cancer progression: total amount of TAMs and specific phenotype of TAMs identified by functional biomarkers. We summarized the data generated in the wide range of international patient cohorts on the correlation of TAMs with clinical and pathological parameters of tumor progression including lymphatic and hematogenous metastasis, recurrence, survival, therapy efficiency. We described currently available biomarkers for TAMs that can be measured in patients’ samples (tumor tissue and blood). CD68 is the major biomarker for the quantification of total TAM amounts, while transmembrane receptors (stabilin-1, CD163, CD206, CD204, MARCO) and secreted chitinase-like proteins (YKL-39, YKL-40) are used as biomarkers for the functional TAM polarization. We also considered that specific role of TAMs in tumor progression can depend on the localization in the intratumoral compartments. We have made the conclusion for the role of TAMs in primary tumor growth, metastasis, and therapy sensitivity for breast, colorectal, lung, ovarian, and prostate cancers. In contrast to other cancer types, majority of clinical studies indicate that TAMs in colorectal cancer have protective role for the patient and interfere with primary tumor growth and metastasis. The accumulated data are essential for using TAMs as biomarkers and therapeutic targets to develop cancer-specific immunotherapy and to design efficient combinations of traditional therapy and new immunomodulatory approaches.

## Introduction

Tumor-associated macrophages (TAMs) are key innate immune cells in tumor microenvironment (TME) that regulate growth of primary tumors, antitumor adaptive immune response, tumor angiogenesis, extracellular matrix remodeling, intravasation in the vasculature, extravasation in metastatic sites; they establish beneficial conditions for metastatic cells in the secondary organs, and interact with various types of therapies (1, 2). Signaling, epigenetic and metabolic mechanisms cooperate to form functional TAM phenotypes (3).

TAMs represent the major component of the innate immune system in TME and can constitute up to 50% of the tumor mass (4). Two main directions of polarization of TAMs can be defined—classically activated M1 (main markers—HLA-DR, CD80/86) and alternatively activated M2 (main markers—CD206, CD163, CD204, stabilin-1) phenotypes (1, 2, 5) (**Table 1**). These typical M2 markers are surface receptors that are responsible for the non-inflammatory clearance of microenvironment from apoptotic bodies, ECM components, soluble mediators of activation of cancer cells and angiogenesis (6–12). In addition to scavenging function (10, 11, 13), stabilin-1 acts as an intracellular sorting receptor that targets chitinase-like proteins SI-CLP and YKL-39 to the secretory pathway (14–19). SI-CLP and YKL-39, in turn, regulate monocyte recruitment and angiogenesis (15, 17, 18, 20).

**Table 1:** Variety of TAM markers in cancer.

Macrophage marker	Function	TAM subpopulation	Type of cancer	Method of detection
CD68	Transmembrane glycoprotein	General macrophage marker	Breast, colorectal, lung, ovarian, prostate	IHC, flow cytometry
CD80	Immunoglobulin superfamily	M1	Colorectal, lung	IHC
CD163	Scavenger receptor for the hemoglobin–haptoglobin complex	M2	Breast, colorectal, lung	IHC, IF
CD204 (MSR1)	Macrophage scavenger receptor	M2	Breast, colorectal, lung, prostate	IHC
CD206	Mannose receptor and C-type lectin	M2	Breast, colorectal, ovarian, prostate	IHC, RNA-seq, flow cytometry
B7-H4	Costimulatory protein of antigen-presenting cells	Not specified	Ovarian, lung	IF, flow cytometry
CCL8 (MCP2)	Monocyte chemoattractant protein	M2	Breast	RNA-seq, qPCR
COX-2	Enzyme responsible for formation of prostanoids	M2	Breast, ovarian	IHC, multiplex IF
HLA-DR	MHC class II cell surface receptor	M1	Lung, ovarian	Multiplexed IHC, IHC
IGF1	Anabolic hormone	M2	Ovarian	Gene chip analysis
iNOS	Enzymes catalyzing the production of NO from L-arginine	M1	Lung, ovarian	IHC and IF analysis
MARCO	Class A scavenger receptor	M2	Lung	Multiplex IF, RNA-seq
MMP-9	Matrix metalloproteinase	M2	Breast, lung	IF
mTORC2	Rapamycin-insensitive protein complex	Not specified	Colorectal	IF
PD-L1 (CD274)	Immunosuppressive protein	Not specified	Ovarian	IF
SIGLEC1 (CD169)	Sialic binding receptor	M2	Breast	RNA-seq, qPCR
SPP1 (Osteopontin)	Protein involved on angiogenesis and metastasis	Not specified	Lung	IHC
Stabilin-1 (RS1)	Scavenger receptor	M2	Breast, colorectal	IHC, IF
TIE2	Angiopoietin receptor	Not specified	Breast	IF
TREM-1	Receptor, regulate inflammatory response	Not specified	Lung	IF, ELISA, Western blot
VEGF	Growth factor	Not specified	Colorectal, ovarian	IHC, qPCR
VSIG4	Costimulatory protein of antigen-presenting cells	Not specified	Lung	IF
YKL-39 (CHI3L2)	Chitinase-like protein, pro-angiogenic and monocyte chemoattractant	M2	Breast	IHC, qPCR
YKL-40 (CHI3L1)	Chitinase-like protein, pro-angiogenic	M1	Breast, lung, prostate	IHC, qPCR, ELISA
ZEB1	Transcription factor – driver of epithelial-mesenchymal transition	M2	Ovarian	IHC

All details and references are presented throughout the text. IHC, immunohistochemistry; IF, immunofluorescence; qPCR, quantitative polymerase chain reaction; TAM, tumor-associated macrophage.

It is commonly accepted that M1-like macrophages exhibit antitumor activity contributing to the activation of adaptive immune response and inflammation, while M2-like macrophages, in contrast, suppress immune function in tumor microenvironment, induce angiogenesis, and support tumor growth and metastasis (21). However, this nomenclature is based on the *in vitro* phenomenon and only schematically reflects vectors of the macrophage polarization *in vivo*, including their polarization in the complex TME. In each cancer type, TAMs can have cancer-specific phenotypes, and can be represented by the heterogeneous populations. Moreover, TAM subtypes can be distinct in various intratumoral compartments, for example in tumor nest and in tumor stroma. Individual TAM phenotypes can be defined by set of markers that not necessarily give clear classification into the M1 and M2 subtypes. The most common histological markers of macrophages belong to the class of transmembrane receptors (mostly of scavenger type); however, new biomarkers that belong to classes of cytokines, growth factors, enzymes, transcription factors, and chitinase-like proteins allow more precise phenotypic and functional characterization of TAMs (**Table 1**).

TAMs originate from two major sources—tissue-resident macrophages and circulating monocytes recruited in tumor cite by growth factors and chemokines, such as M-CSF, CCL2, and CCL5 (21). Local resident macrophages can recognize cancer cells, and it is believed that they have intrinsic ability to eliminate sporadically transformed cells. Different origin can define TAM diversity in the TME. Transformed cells can escape local innate immune control and give origin to cancer cell clones that efficiently recruit monocytes from blood circulation and reprogram resident TAMs. The number of experimental model systems demonstrated that growing tumor can program resident and recruit macrophages to support tumor progression (22, 23). Both monocyte-derived macrophages and resident macrophages (of adult hematopoietic or embryonic origin) accumulate within an expanding tumor (24, 25). Recent study demonstrated that tissue-resident interstitial macrophages in mouse lungs contribute to the pool of TAMs and support tumor growth *in vivo*, while monocyte-derived TAMs contribute to tumor progression in the form of metastasis (26). Interestingly, chemotherapeutic treatment resulted in depletion of both resident and monocyte-derived macrophages, but monocyte-derived macrophages were able to recover and provided phagocytosis-mediated tumor clearance (26). However, not all tumors can do it efficiently, and monocytes and macrophages can also retain their ability to recognize tumor as an unwanted-self structure and inhibit its growth and spread (27, 28). In mouse model of ovarian cancer, CD163+ Tim4+ macrophages from omentum, which have embryonic origin and are uniquely independent of bone marrow-derived monocytes, contributed significantly to the metastatic spread (29). Depletion of CD163+ resident macrophages in tumor-bearing mice with lipid nanoparticles reduced tumor growth and progression (29). We can hypothesize that TAM heterogeneity is defined both by their high plasticity and by their origin from independent specific lineages. The contribution of each of these factors in the final tumor-specific TAM heterogeneity is a highly relevant topic for the investigation.

TAM diversity reflects and defines their specific role in different cancers. A number of studies demonstrated that high infiltration of TAMs in human tumors is associated with poor clinical outcome (1, 2). However, the role of TAMs in tumor growth, lymphatic and hematogenous metastasis and treatment outcomes is specific for each type of cancer. By studying patients, the role of TAMs cannot be defined by loss-of-functions and gain-of-function experimentation, and correlation of TAM amounts, their intratumoral localization and functional phenotypes with clinical parameters is a primary source to draw the conclusion. Therefore, precise definition and accurate selection of clinical parameter are essential. Lymphatic and vascular invasions, characterized by cancer cells’ presence within a definite, endothelial-lined space, are parameters that are potential indicators of the ability of cancer cells to metastasize to the lymph nodes and blood vessels, respectively (30, 31) Vascular invasion may reflect the risk of recurrent disease and prognosis (30). There are survival rates that define the probability of the appearance of one or more of tumor progression parameters. For example, progression-free survival (PFS) is calculated as a period of time between the dates of diagnosis and earliest progression (local recurrence or distant metastasis or death) or last follow-up for patients without progression (32). Similarly, disease-free survival (DFS) is a period of time between the dates of treatment of definite cancer and any signs or symptoms of that cancer; overall survival (OS)—the period where patients still alive for a certain period of time after they were diagnosed with or started treatment for a cancer (33).

In this review we summarize the data about TAM correlation with clinical parameters of widely distributed, dangerous and frequently metastasizing types of cancer: breast, colorectal, lung, ovarian, and prostate. We analyzed the role of TAMs in primary tumor growth and metastasis, and the role of TAMs in the tumor response to therapy with particular focus on tumor relapse and metastatic outbreak. We focus not only on the total amount of TAMs in tumor mass, but we made an accent on the functional TAM biomarkers that can be also distinct in different tumor types.

## TAMs and Breast Cancer

Breast cancer (BC) is the leading cause of cancer-related female deaths in the world. More than 2 million female breast cancer cases have been diagnosed in 2018 worldwide that led to 630,000 deaths (34). Breast cancer is the most studied malignant disease; many diagnostic and therapeutic approaches have been developed for BC patients, and there are a number of ongoing clinical trials. Due to improved treatment and earlier detection, the mortality rate has decreased in most Western countries in recent years, especially in younger age groups (35). The diagnosis of breast cancer is based on the staging system, which, apart from purely anatomical information (tumor, node, metastasis), includes also prognostic information related to tumor biology such as tumor grade, estrogen receptor (ER), progesterone receptor (PR), human epidermal growth factor receptor 2 (HER2), and gene expression data if available (36). Metastatic BC remains virtually an incurable disease with a median overall survival (OS) of around 3 years and 5-year survival of only 25% (37). The most common first metastatic site is the bone, followed by lung, brain, and liver (38, 39). Breast cancer metastasizes also through the lymphatic system to the regional lymph nodes defined as locoregional metastasis (40).

Breast cancer comprises five molecular subtypes that have distinct prognosis and treatment strategies. These five subtypes include: luminal A (ER+, PR+, Ki67 < 20%), luminal B (ER+, PR+ or PR-, Her2+ or Her2-, Ki67 > 20%), triple-negative (ER-, PR-, HER2-), and HER2-enriched breast cancer (ER+, PR+), HER2+) (41). The absence of receptors on the surface of tumor cells of breast cancer is one of the signs of aggressive status and poor prognosis (42). The most aggressive subtypes include HER2 neu-positive and triple-negative breast cancer (TNBC) (42).

BC is characterized by intratumor heterogeneity which is important for disease prognosis and therapy efficacy (43, 44). This is one of the essential difference between human tumors and mouse models, where tumor is mostly homogenous and does not reflect intratumor structures in patients. There are various approaches to describe intratumor morphological and functional heterogeneity. One of these approaches is based on the distinguishing between tumor nest (TN) and tumor stroma (TS) (45). Macrophage infiltration in TN is defined as the tumor-infiltrating macrophages within epithelial cancer cells; TAMs in TS were located in fibrous tissue surrounding the tumor nest (45).

Another approach identifies five intratumor morphological structures based on morphology of cancer cells: tubular, alveolar, solid, and trabecular structures, and discrete groups of tumor cells (44, 46). The level of morphological heterogeneity is distinct in five different molecular subtypes of breast cancer. Tumors with the presence of all five morphological structures were most frequently identified in luminal subtype in comparison with TNBC (47). TNBC was characterized by minimal out of all BC tumor intratumor heterogeneity and frequent presence of only one morphological structure (47). It was demonstrated that breast tumors with alveolar and trabecular structures often demonstrate increased risk of lymph node and distant metastasis, poor response to neoadjuvant chemotherapy (NAC), and decreased metastasis-free survival (48, 49). The distribution of macrophages varied within these morphological structures. CD68 expression was found in TME only of alveolar and trabecular structures and was absent in solid, tubular, and discrete groups (50). Gene expression of SI-CLP, CD206, and Stabilin-1 was also differentially distributed within distinct morphological structures (50).

One more classification of heterogeneity in BC is based on the level of the stromal–parenchymal interactions (51–53). In human breast cancer five distinct morphological compartments characterized by the interaction of tumor cells and immune component can be defined: areas with soft fibrous stroma; areas with coarse fibrous stroma; areas of maximum stromal and-parenchymal relationship; parenchymal elements, and gaps of ductal tumor structures (52, 53). Accordingly, TAM infiltrate localized in specific intratumor compartment or in certain molecular subtype of BC has a different clinical value in patient prognosis. The correlations of TAMs in distinct compartments with parameters of breast cancer progression are discussed below.

### TAMs in Breast Tumors and Metastasis

**Figure 1:**
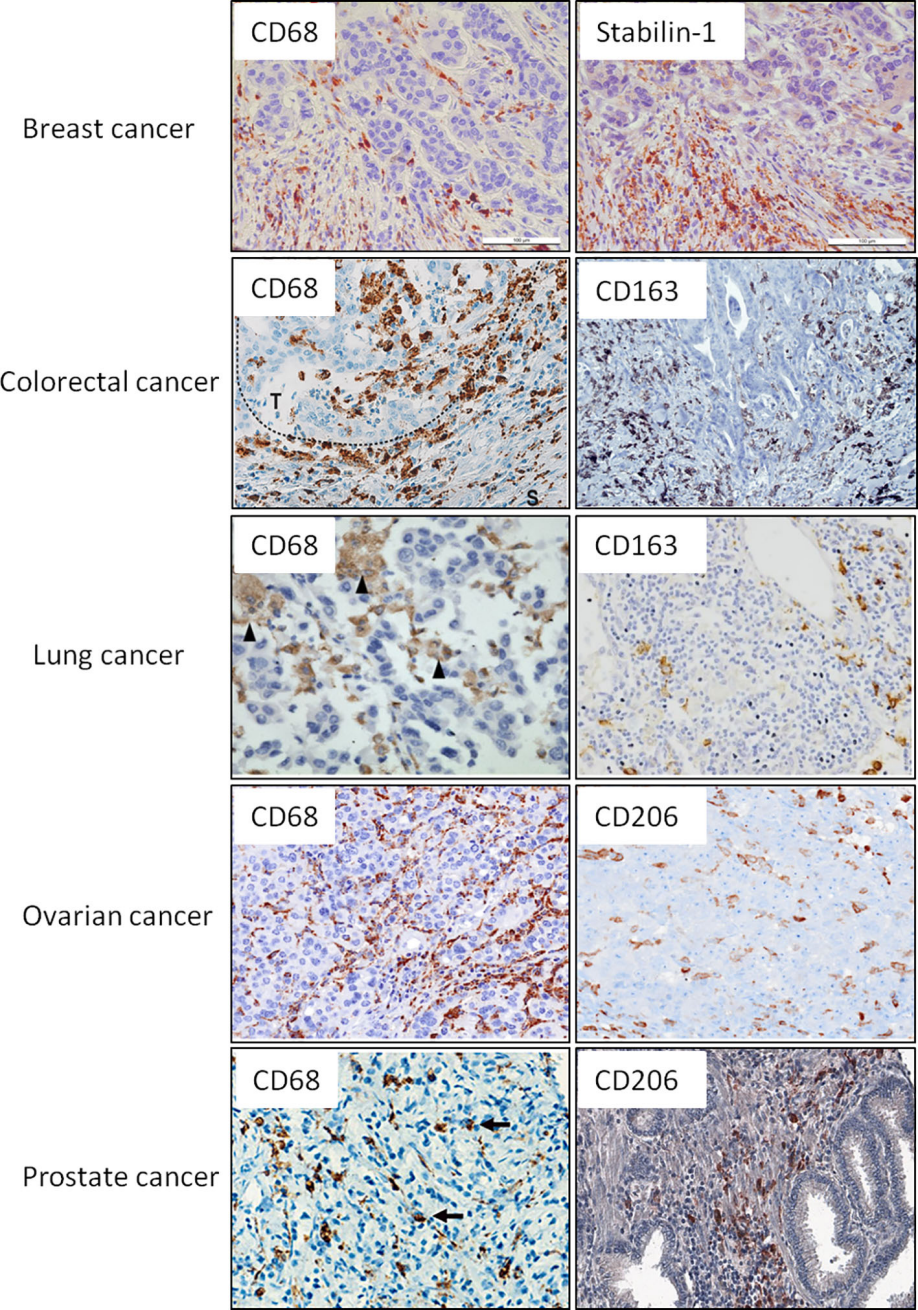
Representative IHC images for the intratumoral macrophages that express CD68 as general macrophage marker and selected M2 markers. Examples of CD68 and M2 markers (CD163, CD206, stabilin-1) are presented for breast, colorectal, lung, ovarian, and prostate cancers. These examples are reproduced from the following publications: for breast cancer (9); colorectal cancer (54, 55); lung cancer (56, 57); ovarian cancer (58); prostate cancer (59). Image for CD206 expression in prostate cancer was kindly provided by Dr. K. Danilko, Bashkir State Medical University. For all published images copyright licenses have been obtained from the publisher.

Two main parameters are used to analyze the clinical significance of TAMs in human cancers—the amount of TAMs defined most frequently by CD68 expression and phenotype of macrophages, defined by different specific M1 and M2 markers (**Figure 1**, **Table 1**).

Breast cancer was the first cancer type in which the tumor-supporting role of TAMs was demonstrated in various animal models (60). One of the first studies demonstrating the negative role of TAM infiltration in the pathogenesis of BC was the immunohistochemical analysis (IHC) of 101 invasive breast carcinoma samples (England, 1996) (61). In this study in a univariate Cox proportional hazard model, increased CD68+ macrophage count was a significant indicator of reduced relapse free survival (RFS) and reduced overall survival (OS) (61). Extensive experimental and clinical data, performed in European, American and Asian cohorts of patients, confirmed the importance of TAM infiltration in the pathogenesis of breast cancer and will be discussed below.

Most of the studies of the amount and phenotype of TAMs in human tumor tissues were performed by using IHC analysis. A number of studies showed that the increase in TAM number, defined by the expression of pan-macrophage marker CD68 correlated with a greater degree of severity of the tumor process (**Table 2**). Thus, the results of meta-analysis of 16 studies (Chinese, Finnish, Swedish, Korean, UK, and USA cohorts) with a total 4,541 BC patients indicated that breast cancer with high TAM infiltration was significantly correlated with characteristics of aggressive biological behavior, such as tumor size, histological grade, ER and PR status, basal phenotype, vascular invasion (68). This meta-analysis showed that high TAM infiltration was not found to be associated with lymph node status (N0 *vs.* N1-3) and HER-2 status (68). Several clinical studies performed on Chinese cohorts of patients with breast cancer demonstrated the association of increased stromal infiltration of CD68^+^ macrophages with larger tumor size, higher histological grade, hormone receptor negativity in BC patients (45, 65). High numbers of CD11c+ macrophages in tumor stroma were associated with a larger tumor size in 367 primary BC patients from the Korean cohort (66) (66). Recent study of 60 primary BC specimens obtained from the Egyptian cohort of patients showed that high CD68+ stromal TAMs significantly correlated with nodal metastasis and vascular invasion (62). In a retrospective study of 1,579 breast cancer specimens (Chinese cohort), high density of both CD68+ TAMs significantly correlated with lymph node metastasis (65).

**Table 2:** Representative studies demonstrating the association of TAMs with tumor progression parameters in breast cancer.

Cohort of patients	Method of detection	TAM correlation with tumor growth and stage	TAM correlation with lymphatic and hematogenous metastasis	TAM correlation with survival	Reference
101 patients with invasive breast carcinoma (UK)	IHC (Chalkley point array)	Not studied	Not studied	Increase of CD68+ TAM amount above the median (12 per HPF ×250) correlates with relapse up to 3 times and with reduced OS rate by 25%	(61)
60 primary BC (Egypt)	IHC (manually)	Increased stromal CD68+ TAM amount above the median (35.3 per hot spot ×400) is indicative for larger tumor size (>5 cm)	Increased stromal CD68+ TAM amount above the median (35.3 per hot spot ×400) correlates with LN metastasis and vascular invasion	Not studied	(62)
371 patients with invasive BC (USA)	Multiplex-IF in TMA (digital imaging scanning)	Presence of CD68+ TAMs positively is associated with tumor size, tumor grade and stage	Not studied	High amount of CD68+ (defined as score 3) and CD163+ (score 3 and 4) TAMs in tumor stroma correlates with reduced OS rate by 20%	(63)
278 BC patients (Finland)	IHC (manually)	Increase of CD68+ TAM amount above the median (34 cells per hot spot ×400) is indicative for histological grade 3. Increase of CD163+ TAM amount above the median (26 cells per hot spot ×400) is indicative for large tumor size and grade 3	High amount of CD163+ TAMs (>26 per hot spot ×400) correlates with LN positivity	High amount of TAMs (CD68+ >34 and CD163+ >26 cells per hot spot ×400) correlates with reduced OS rate by 25%	(64)
1,579 non-metastatic BC (China)	IHC (manually)	Increase of CD68+ and CD163+ TAM amount above the medians (33 and 21 cells, respectively, per HPF ×400) is indicative for histological grade 3	High amount of CD68+CD163+ TAMs (>21 per hot spot ×400) correlates with positive LN status	High amount of CD163+ TAMs (>21 cells per HPF ×400) correlates with reduced OS rate by 10%	(65)
367 non-metastatic primary invasive BC (South Korea)	IHC in TMA (manually)	1.5-fold increased amount of CD68+ and twofold increased amount of CD163+ TAMs are indicative for tumors of grade 3 *vs.* grades 1–2	Not significant	High amount of CD68+ TAMs (>33 cells per HPF ×400) in tumor nest correlates with reduced OS and DFS rates by 20%	(66)
149 patients with invasive ductal carcinoma (Japan)	IHC (not specified)	High TAM density (>190 CD68+ cells/mm^2^, >145 CD163+ cells/mm^2^ and >200 CD204+ cells/mm^2^ per HPF ×200) is indicative for histological grades 2 and 3	Not significant	Increase of CD204+ TAM density over 200 cells/mm^2^ correlates with reduced RFS, distant RFS and DSS rates by 25, 40 and 20%, respectively	(67)

BC, breast cancer; DFS, disease-free survival; DSS, disease-specific survival; HPF, high-power field; IF, immunofluorescence; IHC, immunohistochemistry; LN, lymph node; TAM, tumor-associated macrophages; OS, overall survival; RFS, recurrence-free survival; TMA, tissue microarray.

The amount of CD68+ macrophages in tumor stroma in different cohorts of patients (Chinese, Finnish, Swedish, Korean, UK, and USA cohorts) was an independent prognostic factor for reduced OS, DFS, and RFS of patients with breast cancer (45, 63, 68–71) (**Table 2**). In the two independent cohorts (totaling 677 patients) the presence of CD68^high^/CD4^high^/CD8^low^ signature in tumors was found to be an independent predictor of decreased OS and RFS (72).

#### Subpopulations of TAMs in Breast Cancer Progression

The role of TAMs in the pathogenesis of cancer depends on their phenotype and functional polarization (23). A number of experimental studies *in vitro* and in mouse models demonstrated that M2-polarized macrophages in breast cancer stimulate proliferation of cancer cells, mediate immunosuppression, and induce angiogenesis (73). Major pro-tumor activity of TAMs was demonstrated in PyMT mouse mammary cancer model where TAMs promoted angiogenesis and vascular remodeling in tumors, while macrophage depletion inhibited the angiogenic switch and tumor growth (74). Experimental data correlate very well with the clinical studies demonstrating a supportive role of M2-like TAMs in tumor progression in patients.

Most commonly used M2 markers for the analysis of TAM phenotype in BC include CD163, CD206, CD204, stabilin-1 (**Tables 1**, **2**). Additional markers, expressed also on other cell types, were used to characterize functional TAM phenotype—CD47, COX-2, MMP9, TIE2, YKL-39, YKL-40, PD-L1 (**Table 1**).

Clinical studies showed that CD163+ macrophages in tumor stroma positively correlated with poor histological grade, larger tumor size, Ki67 positivity and LN metastasis in patients with BC (64, 65, 69). A lot of studies from different cohorts of BC patients indicated CD163+ macrophages are predictors of poor survival. Exome-capture RNA sequencing data from 50 primary breast tumors (USA cohort) and their patient-matched metastatic tumors in brain, ovary, bone and gastrointestinal tract revealed that CD163+ macrophages were significantly more abundant in metastatic sites compared to primary tumors primary tumors (75). High amount of intratumor CD163-expressing TAMs, identified by flow cytometry in BC patients from a French cohort, was predictive for reduced survival (76). In a Finnish cohort of 278 BC patients high numbers of both CD163+ and CD68+ cells were associated with short OS of the patients (64). CD163 can be an independent macrophage biomarker indicating poor prognosis for breast cancer patients. Thus, in a study of 371 invasive breast carcinoma specimens from a USA cohort of patients, multivariate analysis revealed that high expression of stromal CD163 is an independent predictor of poor patient OS (63). In this study, the absence of quantitative parameters such as threshold numbers that were used to characterize the expression pattern of CD68 and CD163 in each quartile can potentially be a source of misunderstanding and finally contribute to reproducibility issues (63). In a Chinese study, which enrolled 1,579 non-metastatic BC specimens, CD163+ TAMs but not CD68+ TAMs were associated with poor OS (65), that might be related to the origin of TAMs. IHC analysis of 367 primary invasive BC specimens obtained from patients of a Korean cohort without hematogenous metastasis showed that CD163+ macrophages in tumor nest were an independent prognostic marker of reduced OS and DFS (66).

CD206 is the first identified marker of alternatively activated macrophages, that is induced by IL-4 and used as most specific M2 marker (77). In tumors, CD206 is frequently used to identify switch of TAM phenotype in response to new therapeutic agents and antitumor approaches in experimental models; however, CD163 is predominantly used as M2 markers in clinical studies. Thus, CD206 (M2) macrophages were significant predictor of lower PFS in patients from different racial groups (Latinas and Caucasians) (32).

Specific role of CD204 was found in the Japanese cohort, where high number of CD204+ but not CD68+ or CD163+TAMs was associated with worse relapse-free survival and breast cancer-specific survival (67). However, data about the specific prognostic value of CD206 and CD204 for BC patients is still limited.

Combinations of markers can be also used to identify correlations of TAM amount/phenotype with clinical parameters and metastatic potential BC. For example, the high number of CD68+/COX-2 TAMs in the tumor stroma (TS) and high number of COX-2/CD163 in both tumor nest (TN) and TS were observed in tumors of patients with poor survival that was demonstrated by using multiplex immunofluorescence (63). High expression of MMP-9 in the CD68+/CD163+ TAMs was associated with worse OS in ER^+^ tumors (78). High expression of CCL18+ and SIGLEC1+ TAMs (markers identified by RNA-seq) in 456 breast cancer (USA) was significantly associated with shorter disease-specific survival (DSS) (79). It was noted that TIE2+/CD31+ subpopulation of macrophages abundantly infiltrated metastatic LNs from human breast cancer biopsies but not reactive hyperplastic LNs (80). On the other hand, the amount of stabilin-1+ (M2 marker) TAMs in human breast cancer was mostly abundant on stage I disease (9).

#### TAMs in Different Tumor Compartments Are Differentially Associated With Breast Cancer Progression

The importance of TAM localization within different compartments of the tumor for BC pathogenesis was demonstrated in several studies. The localization of TAMs in tumor stroma (TS) and tumor nest (TN) showed controversial clinical value of TAMs in tumor progression and prognosis (62). Thus, high CD68+ TAMs infiltrating TS were significantly associated with larger tumor. High CD68+, and CD163+ TAM density in TS was significantly associated with LN metastasis (62). Positive correlation with OS was identified for CD68+ macrophages infiltrating TS, but not TN and for CD163+ macrophages in TN and TS structures (63, 69). Interestingly, high expression levels of CD68+ TAMs in the tumor core were significantly associated with shorter OS at the 10-year follow-up while CD68+ TAMs in the tumor periphery were not significantly associated with OS (70). Infiltration of higher number of CD11c+ macrophages in TS was higher in cases with favorable OS, but infiltration in TN did not correlate with OS (66). In the same study the infiltration of higher numbers of CD68+ or CD163+ macrophages in tumor stroma in BC patients didn’t depend on the OS, while infiltration in tumor nest was higher in patients with unfavorable OS (66). For metastatic BC patients, the numbers of CD163+ macrophages in tumor nest were an independent prognostic marker of reduced OS and DFS (66).

The importance of TAM localization in different compartments of tumor was confirmed in several studies of Russian cohort of patients. Our studies demonstrated that in patients with lymph node metastasis the amount of CD68+ macrophages in ductal gaps was lower compared to metastasis-free patients (53). Based on the intratumor morphological heterogeneity the high number of CD68+stabilin-1+ macrophages in solid structures estimated by immunofluorescent analysis was associated with an increased frequency of LN metastasis in luminal B HER2- BC (50). Solid structures demonstrated an elevated expression of factors involved in the mesenchymal type of collective cell invasion (81). So, CD68+stabilin-1+ TAMs localized in solid tumors potentially may contribute to the invasion and the induction of epithelial–mesenchymal transition (EMT) (50).

As was mentioned above, TAMs can be strongly associated with the features of BC molecular subtypes. However, presented results are somehow controversial. Thus, high CD68+TAM infiltration in triple-negative breast cancer (TNBC) had a significantly higher risk for developing distant metastasis and lower rates of DFS and OS (82). In TNBC patients, high CD163+ TAM infiltration and low level of E-cadherin were independent prognostic factors of OS and DFS (83, 84). Oppositely, the analysis of TAMs in 200 cases of basal-like BC (which is similar to TNBC) showed that increased stromal infiltration of CD68+ or CD163+ macrophages correlated with higher 5-year recurrence and 5-year breast cancer mortality (45).

A high level of infiltration of intratumor CD68+ TAMs was an independent prognostic factor for poor DFS in the hormone receptor-positive subgroup, but not in the hormone-receptor negative subgroup (85). At the same time, tissue microarray (TMA) of samples with BC revealed that CD68+ macrophage infiltrates were independently associated with improved RFS for patients with ER-negative tumors (86). In contrast, poor OS correlated with high expression of CD68 in ER^−^ cases, while high expression of CD163 was associated with improved OS in ER^−^ cases but not in ER^+^ cancers (78).

In Swedish, Norway, Chinese, and Egyptian cohorts of patients, CD163+ macrophages positively correlated with estrogen and progesterone receptor negativity, triple-negative/basal-like breast cancer and inversely correlated with luminal A breast cancer (62, 66, 69, 87). Association between high density of CD163+ TAMs and hormonal receptor negativity was also revealed in a meta-analysis of 1,672 specimens of non-metastatic invasive BC (65).

In common, higher infiltration of TAMs, expressed both pan-macrophage marker CD68 and specific M2 markers, is associated with more aggressive molecular subtypes of breast cancer. Taken together, TAM abundance correlated with unfavorable clinicopathological features and survival in patients with breast cancer. Their polarization and localization in different tumor compartments should be taken into account for determining the prognostic and/or predictive role of TAMs.

### TAMs and Breast Cancer Treatment

Treatment of breast cancer is multimodal and includes surgery, radiation therapy, chemotherapy, and molecular treatments (88). Choice of therapy depends on individual course of the disease, including lymph node involvement, hormone receptor status, HER2 overexpression, and patient age and menopausal status. For HER2-positive patients, trastuzumab, an anti-HER2 monoclonal antibody, demonstrates improvement of the survival and administered in combination with chemotherapy. Patients with ER- or PR-positive breast cancer receive endocrine therapy, such as an aromatase inhibitor and selective modulator of estrogene receptors (tamoxifen) (89). For patients with high-risk disease, chemotherapeutic treatment includes an anthracyclines and taxanes, while for low-risk disease, anthracyclines are more commonly used (90). TNBC, the most aggressive type, including BRCA ½ positive patients, should be treated with platinum-based chemotherapy (carboplatin or cisplatin) in neoadjuvant regime which showed more advantages in comparison with standard schemes (91). The most important parameter for the assessment of successful chemotherapeutic treatment and improved survival is the achievement of a pathologic complete response (pCR) (92). After therapy, tumor relapse can happen in up to 40% of patients with breast cancer (93). In case of TNBC, only 30–45% of patients can achieve pCR compared to patients with ER-positive tumors (94). Below we describe how TAMs correlate with different types of therapy and show the perspectives of TAM targeting (**Table 7**).

The accumulation of TAMs in breast tumors after neoadjuvant chemotherapy (NAC) was identified both in animal models and in analysis of different patient cohorts (72, 95). In a study of 311 BC patients of Swedish cohort flow cytometry analysis revealed higher percentage of tumor-infiltrating CD45+CD11b+CD14+ macrophages from women who received NAC (paclitaxel and fluorouracil–doxorubicin–cyclophosphamide) compared to the tumors from women treated with surgery alone (72). In a small cohort of patients (seven patients, USA) who received paclitaxel-based NAC the amount of CD68+ TAMs in the tumor after NAC was higher than in biopsy specimens obtained before NAC (95). Increased accumulation of TAMs after paclitaxel (PTX) treatment was detected also in tumors of PyMT-mice (95).

Predictive value of macrophages for the response to chemotherapy is still controversial. Using CIBERSORT algorithm to summarize the results of 56 studies, totaling 10,988 cases of breast cancer, it was found that M2 macrophages are strongly associated with a lack of pathological complete response (pCR) and resistance to chemotherapy (96). Positive correlation of low CD68 expression with pCR was shown in patients with BC who received trastuzumab in NAC regime (97). Gene chip analysis revealed that high CD68*/*CD8 ratio is also a predictive biomarker for reduced rate of pCR in 311 breast cancer patients from a Swedish cohort that underwent neoadjuvant chemotherapy (PTX and fluorouracil-doxorubicin-cyclophosphamide) (72). In contrast, in 108 patients with BC (UK cohort) who received NAC (capecitabine plus docetaxel preceded by adriamycin and cyclophosphamide), high levels of CD163+ TAMs significantly correlated with a pCR both in tumor and metastatic axillary LNs (98). However, no correlation was found between CD68 expression and pCR (98) (**Table 7**). The semiquantitative method applied in this study for immunohistochemical analysis is useful for description of intergroup differences in CD68+ and CD163+ expression; however, it cannot guarantee the reproducibility of tissue scoring in further studies (98). It can be also hypothesized, that CD163+ TAMs differ in their origin from other CD68+TAMs.

We recently analyzed the predictive role of new TAM-released pro-angiogenic and monocyte chemotactic factor YKL-39 in patients who received PTX- or taxotere-based NAC (17). We found that high gene expression of YKL-39, in biopsies obtained before NAC, positively correlated with increased risk of distant metastasis and poor response (stabilization or progressive disease) to therapy (17) (**Table 7**). In our other study that included 68 female patients with BC (Russian cohort) who received anthracycline-containing NAC, the absence of clinical response is associated with the presence of M2+ macrophage phenotype (YKL-39-CCL18+ or YKL-39+CCL18−) (20). In our study of patients who underwent neoadjuvant chemotherapy (multiple schemes) CD68+ TAMs in areas with parenchymal elements negatively correlated with lymphatic metastasis (52).

In contrast to YKL-39, high epithelial and stromal PD-L1 expression in biopsies obtained before NAC (PTX-based or platinum-based) correlated with increased rate of pCR after NAC, especially in hormone-positive and Her2-postive breast cancer (99).

Several studies in mouse models confirmed the effectiveness of treatment based on the combinations of chemotherapeutic agents and inhibitors of macrophage activity in tumor. Thus, *in vivo* in MMTV-PyMT (PyMT) tumor-bearing mice, treatment with paclitaxel (PTX) in combination with CSF1 and cKIT receptor tyrosine kinases inhibitor (PLX3397) but not with PTX alone resulted in a significant reduction in CD31+ vessel density, reduced pulmonary metastases, and activation of cytotoxic T cell response (72). Using the same mouse model, it was found that TAMs are the source of the cathepsins during PTX treatment. Combining PTX with cathepsin deletion [by JPM-OEt (JPM), a pan-cathepsin inhibitor] significantly improved therapeutic efficacy of PTX, inhibited tumor growth and metastatic burden, and improved late-stage survival (95). In this study the addition of low-dose cyclophosphamide enhanced antitumor efficacy of treatment (95). In another study using MMTV-PyMT transgenic mice, PTX showed more pronounced antitumor effect in combination with the selective class IIa histone deacetylase (HDACIIa) inhibitor TMP195 which modulates macrophage phenotypes promoting phagocytosis of cancer cells (100).

In mice bearing chemoresistant MCF-7 breast cancer xenograft treatment with combined chemotherapy (CMF—cyclophosphamide, methotrexate, 5-fluorouracil) and anti-CSF-1 Fab [murinized, polyethylene glycol-linked antigen-binding fragment (Fab) against mouse (host) CSF-1] reversed chemoresistance of MCF-7 xenografts, reduced angiogenesis, macrophage recruitment, suppressed tumor growth, down-regulated expression of the chemoresistance genes, and improved survival rates (101). In cyclophosphamide-treated mouse mammary tumors and in human breast cancer that underwent NAC (cyclophosphamide), the M2 subpopulation of TAMs (CD206+TIE2^hi^CXCR4^hi^) was found around the blood vessels, where they promoted tumor revascularization and relapse (102).

It was found that TAMs mediate the resistance of breast cancer during endocrine therapy by tamoxifen. MCF-7/THP-1 co-injected mice showing more extensive growth were characterized by tamoxifen resistance in contrast to MCF-7-injected animals (103). *In vitro* generated TAMs from THP-1 cells showed M2 phenotype (CD163+) when cultured with conditioned medium from tamoxifen-resistant MCF-7 cell lines (104). The possible mechanism of the resistance is a feedback loop between TAM-released CCL2 and PI3K/Akt/mTOR signaling activated in cancer cells (104). Clinically, in ER-positive and Her2-negative breast cancer, CD163+ TAMs more abundantly infiltrated tamoxifen resistant tissues in comparison with tamoxifen sensitive tissues (105).

Currently, there is no consensus about the effect of TAMs on the efficiency of chemotherapy in patients with breast cancer. However, most of mouse models demonstrated the negative role of TAMs in the tumor response to chemotherapeutic treatment.

## TAMs and Colorectal Cancer

Colorectal cancer (CRC) is the fourth most commonly diagnosed malignancy and the fifth leading cause of cancer-related deaths in the world. In 2018 more than one million new cases of colorectal cancer were diagnosed and almost 550 thousands deaths were registered worldwide (34). Five-year survival of patients with CRC is still below 60% in most European countries (106).

Major pathological parameters used for the prognosis of CRC include TNM stage, microsatellite status tumor grade, and lymphovascular invasion. The mutation status of KRAS, BRAF, and NRAS has a predictive value for the response to anti-EGFR therapy in metastatic context (107). The most common site of metastasis with the worst prognosis is the liver. Other sites of metastasis include the lung, bone, multiple sites, and brain (108).

Similar to breast cancer, colorectal tumors display intratumor heterogeneity that is based on the abnormalities in three different main molecular pathways: (1) chromosomal instability (CIN) (more than 50% of cases), (2) microsatellite instability (MSI) (6–15% of cases), and (3) CpG island methylating phenotype (CIMP), or epigenetic instability (up to 20% of cases) (107, 109).

Although the colon cancer and rectal cancer are usually epidemiologically registered as CRC, they should be considered as two separate diseases due to their topography, surgical challenge, therapy, complications, and relapse pattern (108, 110). Rectal cancer is characterized by more frequent local relapses than colon cancer. Additionally, colon cancer is divided to the left and right cancer types (108). The Consensus Molecular Subtypes (CMS) classification of colon cancer was proposed in 2015 by Justin Guinney and colleagues, who analyzed the data of gene expression of 4,151 colon cancer patients (111). Four types of CMS are proposed: 1) CMS1 (MSI, immune type, 14% of total CRC) is characterized by hypermutation, high microsatellite instability, pronounced immunogenicity, mutations of the BRAF gene; 2) CMS2 (canonical, 37% of total CRC) is an epithelial type characterized by activation of Wnt and MYC signaling pathways and high frequency of copy number changes in somatic cells; 3) CMS3 (metabolic type, 13% of total CRC) is an epithelial type characterized by metabolic dysregulation and mutations of the KRAS gene and by heterogeneous microsatellite and chromosomal instability; 4) CMS4 (mesenchymal type, 23% of total CRC) is characterized by activation of the TGF-*β* signaling pathway, epithelial–mesenchymal transition, severe stromal infiltration, active neoangiogenesis, and poor prognosis. One subtype with mixed characteristics (13% of total CRC) is also distinguished, that can be also a transition phenotype or special case of intratumor heterogeneity (111). Both CMS1 and CMS4, which are immunogenic, showed high levels of infiltrating CD8+ cytotoxic lymphocytes and CD68+ TAMs (112). Stromal cell infiltration was significantly higher in tumors with CMS4 compared to other CMS. In contrast, the canonical (CMS2) and metabolic (CMS3) subtypes with intermediate prognosis exhibit less pronounced immune and inflammatory responses (112). Despite high heterogeneity of CRC, the prognostic role of TAM infiltrate in the context of different molecular subtypes or histological localizations remains to be investigated.

### TAMs in Colorectal Tumors and Metastasis

In colorectal cancer (CRC), a number of *in vitro* studies showed pro-tumor activity of macrophages that induce growth and invasive behavior in colon cancer cells (113–115). For example, human colon cancer cell lines (HCT116, WiDr, SW480, and RKO) co-cultured with monocyte cell lines (THP-1 and U937) showed enhanced invasiveness compared to control tumor cells alone (113). Co-cultured HT-29 or HCT116 colorectal cell lines with TAMs (THP-1 cells stimulated by conditioned media from CRC cell lines) demonstrated enhanced EMT supporting migration, invasion, and circulating tumor cells (CTC)-mediated metastasis. Invasive phenotype of CRC tumor cells was regulated by TAM-derived IL-6 which activated the JAK2/STAT3 pathway and resulted in increased FoxQ1 expression. In turn, the production of CCL2 by tumor cells was enhanced that promoted macrophage recruitment (114). The limitation of these studies was the use of proliferative THP-1 cells which differ significantly from human primary blood monocytes. *In vitro* condition medium (CM) from LPS-treated macrophages containing IL-1b, IL-6, and TNF-α induced proliferation of HCT116 colon cancer cell line, increased NF-kB activity and VEGF secretion in cancer cells (116). In another study, HCT116 and HT29 colorectal cancer cells cultivated with M2 macrophages expressed reduced levels of E-cadherin but increased levels of vimentin and showed enhanced invasive ability (115). It was also found that TAMs can produce ECM proteins (the abundance of collagen types I, VI, and XIV) in CRC, that induce ECM remodeling (117).

In contrast, there is a series of convincing evidence obtained by Beelen R. and Bögels M. that indicates that macrophages in CRC have M1 phenotype with antitumor activity (27, 28, 118). Thus, they found that human monocytes incubated with the conditioned media of colon carcinoma cells (HT29, HCT116, RKO, SW620 and SW948) show high production of pro-inflammatory cytokines (IL-6, IL-12, and TNF-α) and increased gene expression of the chemokine ligand CXCL13 but decreased expression of anti-inflammatory cytokine IL-10 and the pro-angiogenic cytokine IL-8 (27). Human monocytes stimulated with conditioned media of breast carcinoma cell lines (SKBR3, MCF7 and ZR-75–1) stimulated in macrophages enhanced production of IL-10 and expression of mannose receptor 1 (MR1), CCL17, and CCL22, that are M2-associated chemokines (27). In rat model of CRC tumors, administration of flavonoids rutin and luteolin, that reduce monocyte migration, resulted in reduced number of intratumoral ED1+ immature macrophages without affecting ED2+ resident macrophages (28). Rutin and luteolin administration enhanced tumor size and increased peritoneal metastases (28). Incubation of co-culture of BMDMs and CRC cancer cells (CC531s) with MG4-c1, MG4-c2a, or MG4-c2b mAb led to increased tumor cytotoxicity and decreased tumor cell growth (118). In CRC rat model, resident liver macrophages (Kupffer cells) were involved in cytotoxic effect eliminating tumor cells under monoclonal antibody treatment (118).

#### Favorable Role of Total Amount of TAMs in Prognosis of CRC

CD68+ TAMs serve as a good prognostic factor for patients with CRC in different cohorts of patients (**Table 3**, **Figure 1**). Thus, IHC analysis of Japanese cohort of 30 patients with CRC showed that low levels of CD68+ TAMs in invasive front and tumor stroma were associated with more advanced colorectal cancer, while high amount of TAMs was found in patients with good prognosis (126). In European cohorts of patients, similar correlations have been identified. Tissue microarray of 100 patients with colon cancer (Germany) demonstrated that amounts of CD68+ macrophages were decreased at higher stage tumors (127). Analysis of 210 samples with primary colorectal cancer (Bulgaria) showed that lower number of CD68+ TAMs in tumor invasive front significantly correlated with the presence of metastases in local lymph nodes, with distant metastases and with more advanced tumor stage (III and IV stages) (119). Lower number of CD68+ cells in tumor border was also found in patients where tumor cell invaded the blood circulation, lymph vessels or were characterized by perineural invasion and lower grade of inflammatory infiltration. High level of TAM infiltration in tumor invasive front was an independent favorable prognostic factor for overall survival (119). High intraepithelial and stromal expression of CD68 predicted long-term OS and correlated with significantly less tumor budding at the invasive front and absence of lymph node metastasis in the Greek cohort of 201 patients with primary CRC (120). In a Swedish cohort of 488 patients with colon and rectal cancer, high infiltration of CD68+ macrophages was associated with high survival of patients (121). Significant positive association between DFS and CD68+ cells was demonstrated in the USA cohort of 188 patients with colorectal cancer liver metastasis (128).

**Table 3:** Representative studies demonstrating the association of TAMs with tumor progression parameters in colorectal cancer.

Cohort of patients	Method of detection	TAM correlation with tumor growth and stage	TAM correlation with lymphatic and hematogenous metastasis	TAM correlation with survival	Reference
210 patients with primary CRC (Bulgaria)	IHC (digital imaging scanning)	Amount of CD68+TAMs (per hot spot ×320) in invasive front is decreased by almost 25% in advanced III + IV stages (114.9 ± 91.9 *vs.* 150.2 ± 102.3 in I + II stages)	Amount of CD68+TAMs (per hot spot ×320) in invasive front is decreased by 17% in tumors with regional LN metastases (119.4 ± 96.5 *vs.* 143.3 ± 100.0 in cases with negative LN), and by 42% in tumors with distant metastases (150.4 ± 105.8 *vs*. 87.8 ± 54.3 in negative cases)	Increased amount of CD68+ TAMs above 48.6 cell/mm^2^ in tumor stroma and above 105.2 cell/mm^2^ in invasive front is associated with increased OS rates by 10 and 40%, respectively	(119)
201 patients with primary CRC (Greece)	IHC in next-generation TMA (manually)	High amount of intraepithelial CD68+ TAMs (counted per hot spot ×400) predicts less tumor budding High amount of CD163+ TAMs (counted per hot spot ×400) is indicative for G1-2 grades	High amount of CD68+ and CD163+ TAMs is associated with absence of LN metastasis	High amount of CD68+ TAMs correlates with better OS (increase by 40%)	(120)
488 patients with colon and rectal cancer (Sweden)	IHC (manually)	High CD68+ infiltration (defined as grades 3 and 4 in hot spot ×200) at the invasive front is indicative for I+II stages and well-moderate grade	Not studied	High CD68+ infiltration (defined as grades 3 and 4 in hot spot ×200) at the invasive front correlates with increased DSS rate by 30%	(121)
160 patients with stage IIIB and IV colon carcinoma (China)	IHC (manually)	Not significant	High CD68+ infiltration (defined as grades 3 and 4 in hot spot ×200) at the invasive front is associated with absence of hepatic metastasis	High CD68+ infiltration (defined as grades 3 and 4 in hot spot ×200) at the invasive front correlates with increased OS rate by 30% and liver-metastasis free survival rate - by 20%	(122)
163 patients with rectal cancer (Sweden)	IHC in TMA (manually)	Not significant	Not studied	Presence of CD163+ TAMs in tumor tissue is associated with reduced OS and RFS rates by 40%	(123)
81 patients with CRC (China)	IHC (manually using immunoreactive score)	Increase of CD163+ TAM expression above the median (measured semiquantitatively at ×400) is indicative of III TNM stage, poor tumor grade	High CD163+ expression positively correlates with lymphovascular invasion and N2-3 LN status	High CD163+ expression is associated with reduced OS rate by 30% and RFS by 20%	(114)
521 and 314 patients with stage II CRC (China)	IHC in TMA (digital imaging scanning)	Increase of CD206/CD68 ratio ≥ 0.77 is indicative of poor differentiation and undifferentiation status and pathological T4 stage	Increase of CD206/CD68 ratio ≥ 0.77 is associated with lymphatic/vascular invasion and perineural invasion	Increase of CD206/CD68 ratio ≥ 0.77 correlates with reduced DFS rate by 40% and OS by 30%	(124)
159 patients with advanced colorectal cancer (stage IV) (Finland)	IHC (manually)	Not studied	Low amount of intratumoral stabilin-1+ TAMs (<10 cells per ×400 hotspot) correlates with low number of distant recurrences	High amount of peritumoral stabilin-1+ TAMs (≥10 cells per ×400 hotspot) correlates with longer DFS time (103 *vs*. 63 months in cases with low amount) at stages II and III, but correlates with reduced DSS rate by almost 2 times in stage IV patients	(125)

CRC, colorectal cancer; DFS, disease-free survival; DSS, disease-specific survival; IF, immunofluorescence; IHC, immunohistochemistry; LN, lymph node; TAMs, tumor-associated macrophages; OS, overall survival; RFS, recurrence-free survival; TMA, tissue microarray.

IHC analysis of CD68 expression in CRC tissue in Chinese cohorts of patients revealed similar correlations. Thus, a study of 160 patients with stage IIIB and IV colon carcinoma demonstrated that high density of CD68+ macrophages in invasive front of tumor was associated with higher 5-year survival rate and lower hepatic metastasis (122). However, in this study, the exact quantitative parameters have to be interpreted carefully, since the semiquantitative method applied relies on a subjective visual assessment that could affect reproducibility (122). In 521 patients with stage II colon cancer after radical resection, low CD68+ TAM density was significantly associated with perineural invasion (124). This finding was confirmed by using validation cohorts (314 eligible patients) (124). IHC staining of 118 CRC tissues demonstrated positive association of intratumoral CD68+ TAM count with depth of invasion, lymph node metastasis, and tumor staging. Besides, a significant association between CD68 expression and MMP-2 and MMP-9 expression in CRC was found (113). The difference of this study was the fact that CD68+ TAM infiltration was estimated only in intratumor compartment where they have very low density. For M1 macrophages expressing NOS2, their high infiltration was demonstrated to be significantly associated with improved cancer-specific survival in patients with colon cancer of the Swedish cohort (54).

#### Negative Role of M2-Like TAMs in Prognosis of CRC

In contrast to the total amount of macrophages defined mostly by CD68 marker, M2-like phenotype of macrophages is rather indicative for the negative prognosis of patients with CRC (**Table 3**). IHC analysis of Chinese cohort of 81 patients with CRC showed that high expression of stromal CD163 at tumor invasive front was significantly associated with tumor grade, lymphovascular invasion, tumor invasion, lymph node metastasis, and TNM stage and correlated with poor RFS. High level of CD163 was also associated with reduction of E-cadherin and high expression of vimentin in cancer cells, an indication of EMT (114). In the same cohort high CD163+/CD68+ ratio in the tumor front, but not in tumor stroma, was closely correlated with enhanced lymphovascular invasion, tumor invasion, and TNM stage as well as recurrence-free survival (RFS) and OS of patients with CRC. Moreover, CD163+/CD68+ ratio in tumor front was also significantly associated with EMT program and CTC amount (115). A study of 150 patients of Spanish cohort demonstrated that CD163+ macrophages were found in tumor invasive front while CD80+ cells located in adjacent normal mucosa in less invasive T1 colorectal cancer that was detected by immunohistochemistry. At stage III CRC, higher CD68 and lower CD80/CD163 ratio was associated with decreased OS (129). Tissue microarray of samples obtained from 163 patients with rectal cancer from the South Eastern region in Sweden demonstrated that CD163+ biopsies have earlier local recurrence and poor survival (123, 130). One contradictory study was found. In 201 patients with primary CRC (Greece), improved survival was identified in tumors with strong stromal infiltration of CD163+ M2 macrophages, which presented 40% of the total macrophage population (120). CD163+ macrophages were also predictive of the lower tumor grade and less lymph node metastasis that was demonstrated by next-generation tissue microarray construction (120). In this study, expression scores were dichotomized according to the mean into low and high groups; however, the authors did not provide the information about mean number used as threshold, thereby limiting our ability to compare the obtained results with data from other studies (120).

Using two independent cohorts of Chinese patients with stage II CRC (521 patients and 314 patients) it was found that high CD206+ TAM density was significantly associated with stage II of CRC characterized by poor differentiation (124). A high CD206/CD68 ratio was significantly associated with poor differentiation, pathological T4 stage, lymphatic/vascular invasion, and perineural invasion. Besides, patients with CD206+ TAM density and high CD206/CD68 ratio had significantly worse DFS and OS (124). CD204+ TAMs were abundantly detected in high-grade colorectal adenomas in comparison with low-grade adenomas that was shown immunohistochemically in 88 tubular or tubulovillous adenomas (131). In advanced colorectal cancer (stage IV), patients with a high number of peritumoral or intratumoral stabilin-1+ macrophages had a shorter DSS that was found in the Finland cohort of 159 patients. Moreover, a low number of suppressive intratumoral stabilin-1+ macrophages in this cohort correlated with a low number of distant recurrences (125).

TAMs were also found to be involved in tumor progression by expressing several markers expressed also by other cell types. Interestingly, VEGF+ TAMs/stroma in colon cancer is indicative for the increased survival in comparison with patients with the absence of VEGF expression in stroma (132). Patients with CRC (Swiss cohort) tumors with VEGFA gene amplification have reduced CD68+ and CD163+ TAM infiltration, while high-grade tumors are associated with increased CD163+ and reduced CD68+ macrophage infiltration (55). In another study, high percentage of VEGFR1+ macrophages in lymph node metastasis was associated with worse outcome in patient with CRC (133). VEGFR1+ circulating monocytes in blood of patients with LM predicted reduced PFS and site of recurrence (liver) in CRC (133). In contrast, mTORC2 activity (pPKC*α* staining) in macrophages was negatively associated with tumor stage and LN metastasis in the Austrian cohort of CRC patients. Low mTORC2 activity in macrophages in tumors was significantly associated with lower survival rate (134).

### TAMs and Colorectal Cancer Treatment

The main strategies in the treatment of colorectal cancer are surgery, radiation therapy (or chemoradiation), chemotherapeutic treatment (135). Chemoradiation and short-course radiotherapy have more advantages than chemotherapy alone and result in improved survival. Conventional chemoradiation regimens include fluorouracil or capecitabine. Addition of oxaliplatin to fluorouracil improved DFS (135). FOLFOX (oxaliplatin-containing regimens) and FOLFIRI (irinotecan-containing regimens) showed more efficacy than 5-FU alone (135). Neoadjuvant FOLFOX chemotherapy combined with radiotherapy followed by radical resection is the standard combined therapy in patients with locally advanced colon cancer (136). However, the treatment response to neoadjuvant CRT is variable from a pathological complete response (pCR) to total resistance. pCR was associated with the favorable survival, however, has ranged from 10 to 30% (137).

The presence of activating mutations in the KRAS, NRAS, and BRAF genes is the criterion to refuse the therapy with the anti-EGFR agents. Mutations in these genes occur in about 55–60% of colorectal cancers. Patients with KRAS, NRAS, or BRAF mutations do not benefit from anti-EGFR therapies (138). Targeted drugs, such as bevacizumab (human anti-VEGF antibody), cetuximab, and panitumumab (human EGFR monoclonal antibodies) have been proven to be effective against metastatic CRC in patients (139). Survival of patients with metastatic CRC increased with the addition of irinotecan or oxaliplatin to 5-FU. However, the recurrence rate remains high, especially in rectal cancer.

The role of TAMs in the efficiency of treatment is strictly limited in the studies of patients with CRC (**Table 7**). High CD68+ TAM infiltration in tumor tissue of 123 patients with metastatic CRC decreased the efficacy of bevacizumab plus FOLFIRI scheme (folinic acid, 5-fluorouracil, irinotecan) of chemotherapy (140). In stage II colon cancer with high CD206/CD68 ratio, adjuvant chemotherapy significantly improved the DFS rate from 38.9 to 68.0% at 3 years and from 33.1 to 66.0% at 5 years (124). Oppositely, for 208 patients resected for stage III colorectal cancer, high CD68+ TAMs in invasive front of tumor and in metastatic lymph node were associated with better DFS only in 5-fluorouracil-treated patients compared to untreated ones (141).

Clinical trial of bevacizumab plus FOLFIRI treatment in patients with metastatic colorectal cancer demonstrated that single-nucleotide polymorphisms in genes regulating TAM-related functions significantly associated with clinical outcome in metastatic CRC patients (142). *CCL2 rs4586, CCL18 rs14304*, and *IRF3 rs2304205* correlated with PFS in KRAS mutant patients of the TRIBE cohort; *TBK1 rs7486100* correlated with OS in KRAS wild-type patients of TRIBE cohort (142).

Most pieces of evidence are found *in vitro* or in animal models. In several studies TAMs were found to be involved in the resistance of tumor to 5-fluorouracile (5-FU). Thus, 5-FU treatment significantly increased the infiltration of CD68+TAMs in the mouse subcutaneous CT-26 tumors (143). *In vitro* putrescine (polyamine) secreted by TAMs significantly attenuated 5-FU-induced growth inhibition of SW-480 and HCT-116 cell lines (143). 5-FU treatment induces CCL22 secretion by M2 macrophages *in vitro* (144). Co-culture of colon cancer cells and M2 macrophages treated with 5-FU indicated that macrophages mediate cell migration and invasion in CRC cells inducing EMT and activating PI3K/AKT pathway (144). CCL22 neutralizing antibody increased the apoptosis in cancer cells. Clinically, CCL22 expression was elevated in patients with colorectal adenocarcinoma and was positively associated with CD163+ TAMs. Patients with higher CD163+ M2 macrophages and high expression of CCL22 in CRC tissue had worse overall survival (OS) (144).

Administration of oxaliplatin (OXP) with other potential antitumor drugs demonstrated antitumor effect in several mouse models of CRC. The expression of F4/80 and iNOS significantly decreased under oxaliplatin (OXP) treatment in tumor-bearing mice (145). OXP inhibited the M1-like macrophages polarization while had little effect on differentiation into M2-like macrophages *in vitro* (145). Administration of oxaliplatin combined with Toll-like receptor agonists R848 reversed the functional orientation of MDSCs towards M1-like macrophages and strengthened antitumor effect of oxaliplatin *in vivo* (145). In an abdominal implantation model of colon cancer intraperitoneal administration of OXP inhibits tumor cell growth by a decrease in CD11b+F4/80high macrophages in tumors (146). Treatment of CT26 tumor-bearing mice with combination of oxaliplatin with trifluridine/tipiracil (FTD/TPI), a new antimetabolite agent, induced TAM depletion and promoted CD8+ T-cell infiltration in tumors (147).

Contradictory results were found for cetuximab interaction with macrophages. In AOM/DSS-induced colon cancer mouse model, cetuximab (anti-EGFR antibody) treatment inhibited total F4/80+/CD11b+ TAMs and M2 (F4/80+/CD206+) TAM accumulation (148). Down-regulation of gene expression of M2 polarization markers, ARG1, IL-10, and IL-4, was observed in tumor. *In vitro* THP-1 cells stimulated with conditioned medium from HCT116 cell with EGFR knockdown acquired M1 phenotype (by upregulation of IL-12, CCR7, and TNF-α) and down-regulation of M2-related markers (IL-10, ARG1, CCL17, CCL22, and IL-4) (148). In contrast, cetuximab induced production of anti-inflammatory and tumor-promoting mediators, including IL-10 and VEGF activating M2-macrophages in co-culture of CRC cell line and human monocyte-derived macrophages (149).

In summary, there is still no agreement about the role of TAMs in the treatment of CRC. Such contradictory results clearly depend on the animal model, type of *in vitro* study, patient cohort, and type of anti-cancer drug. Most of presented studies indicate that TAMs enhance tumor resistance to chemotherapy in colorectal adenocarcinoma. Therefore, to achieve the maximum efficiency of chemotherapy in CRC, the combined approaches that include targeting of TAMs should be developed.

## TAMs and Lung Cancer

Lung cancer is the leading cause of cancer-related death and the second most diagnosed cancer worldwide. More than two million new cases and more than 1.7 million deaths were registered in 2018 worldwide (34).

Lung cancer is highly heterogenic and can be localized in different anatomic compartments of the lung and manifests in variable symptoms (34, 150). There are two main histological types of lung cancer: non-small cell lung carcinomas (NSCLC) (85% of patients) and small-cell lung carcinomas (SCLC) (15%). These two types differed by growth, metastatic spread, and treatment strategy. NSCLC is classified into three subtypes: adenocarcinoma, squamous cell carcinoma, and large cell carcinoma (34, 150). Unfortunately, around 70% of patients are diagnosed at the advanced stages of the disease (stage III or IV) (34). Around 40% of the newly diagnosed patients have stage IV of NSCLC (151). The 60-month OS rate for NSCLC remains poor, from 50 to 70% in patients with early-stage operable disease, dropped to 2–5% in patients with stage IVA–IVB (150). The brain is the most frequent site of distant metastasis in lung cancer patients, and metastatic process is a major cause of morbidity and mortality. Brain metastases are found in 80% of SCLC and 30% NSCLC (152, 153).

The lung is one of the major barrier organs for the defense of the organism against foreign particles and pathogens. The lung anatomy and cellular composition are ideal to fulfil this defense function without induction of unnecessary inflammation (77). Numerous components of the immune system, including abundant alveolar macrophages (AMs), are involved in the maintenance of the immunological homeostasis. The role of AMs in lung cancer remains contradictory. Lung tumors activate tumor-supporting role of resident AMs by decreasing their antibody-mediated cytotoxicity and antigen processing and presentation ability and by enhancing their pro-angiogenic activity (154, 155). However, in numerous studies antitumor activity of AMs has also been demonstrated (155). The mechanism of AM programming by TME remains to be investigated.

We focused on TAMs located in lung tumor tissue and discussed the prognostic relevance of TAMs below.

### TAMs in Lung Tumors and Metastasis

In lung cancer TAMs represent the most abundant immune cell component of TME (154) (**Figure 1**). TAMs in lung cancer promote cancer proliferation, epithelial–mesenchymal transition (EMT), invasion and metastasis, resulting in poor patient outcome (156, 157). Lung cancer cells activate macrophages and other non-malignant stromal cells, such as fibroblasts and vascular endothelial cells, in the TME to form a positive feedback between tumor cells and TAMs promoting tumor progression (158–160). However, the detailed mechanisms by which TAMs promote malignancy in lung cancer remain largely unclear.

Numerous studies confirmed that in lung cancer TAMs contribute to tumor progression and metastasis through the production of variety of chemokines and growth factors (156, 161–163). *In vitro* lung carcinoma cells (human NSCLC A549 cells) induce polarization of THP-1 cells to CD206+ M2 phenotype (156). In turn, M2 macrophages promoted EMT and invasion in lung cancer cells upregulating CRYAB expression on tumor cells and activating the ERK1/2/Fra-1/SLUG signaling pathway. Clinically, high expression of CRYAB on tumor cells was associated with lymph node metastasis and tumor stage (III–IV) (156). In human and mouse tumors TAM accumulation correlated with the expression of integrin *α*v*β*3 on cancer cells, a known driver of epithelial cancer progression and drug resistance (164). In mouse model of Lewis lung carcinoma (LLC), macrophage depletion with clodronate in combination with genetic ablation of CCR2 and CX3CR1 (receptors responsible for monocyte recruitment) inhibited cancer cell growth and metastasis enhancing survival in mouse (160). In human lung cancer samples from 72 NSCLC patients, intratumor CD68+ TAM infiltration and CCR2 expression correlated with tumor stage and metastasis (160).

#### Total Amount of TAMs in Lung Cancer Progression

Lung macrophages are major component of lung tissue due to their essential role in the clearance of the infectious and non-infectious contaminants of the air (77). Due to their high abundance, their increased amount is not the critical factor for the progression of lung cancer. However, there are still some reports in Chinese cohorts where the correlation of CD68+ cells with clinical parameters of lung cancer was examined (**Table 4**).

**Table 4:** Representative studies demonstrating the association of TAMs with tumor progression parameters in lung cancer.

Cohort of patients	Method of detection	TAM correlation with tumor growth and stage	TAM correlation with lymphatic and hematogenous metastasis	TAM correlation with survival	Reference
68 NSCLC patients (China)	IHC (not specified)	Positive CD68+ expression correlates with higher TNM stage (III and IV)	Positive CD68+ expression correlates with the presence of LN metastases	Not studied	(165)
160 NSCLC patients (Japan)	IHC (manually)	High stromal (>380/mm^2^ in ×400 HPF) and alveolar CD163+ TAM densities (>400/mm^2^) are indicative for increase of CRP level up to 2 times, increase of invasive size by 20–45%, poor differentiation and advanced stages (II and III)	1,4-fold increase of stromal and alveolar CD163+ TAM densities is indicative for tumors with N1–N3 nodal status *vs*. cases without LN metastases	In early stages (0 and I), high stromal CD163+ TAM density correlates with reduced DFS rate by 20% and OS by 12%. In advanced stages (II and III), high alveolar CD163+ TAM density correlates with reduced DFS rate by 22% and OS by 17%	(166)
335 NSCLC patients (Danmark)	IHC (digital imaging scanning)	Not significant	twofold increase of median area fraction of CD163+ TAMs in tumor nest and 1.5-fold increase in tumor stroma are found in cases with N1/N2 nodal status *vs*. those without LN metastases	Not significant	(167)
297 NSCLC patients (Japan)	IHC (digital imaging scanning)	Increase of stromal CD68+ and CD204+ TAM amounts above the medians (48 and 15, respectively, under ×200) positively correlates with Ib-IV stages and G2-G4 histological grade	High amount of CD68+ (>48) and CD204+ (>15) TAMs correlates with pleural invasion and LN metastasis	High amount of CD68+ (>48) and CD204+ (>15) TAMs in tumor stroma correlates with decreased DFS rates by 10%	(168)
553 primary NSCLC patients (Norway).	Multiplexed-IHC in TMA (digital imaging scanning)	Increase of stromal HLA-DR+/CD68+ TAM amount >1.0 under ×200 is indicative for lower T stages (T1 and T2)	Not studied	High amount of intratumoral and stromal HLA-DR+/CD68+, CD204+ and CD68+ TAMs correlates with increased DSS rates (appr. by 10-20%)	(169)
80 NSCLC patients (Lithuania)	IHC (manually)	High amount of CD163+TAMs is found in tumors with poor differentiation (median 118 per 10 HPFs under ×400) versus moderate and well differentiated (median 108)	High amount of stromal CD68+ TAMs is found in tumors with N1-N3 nodal status (median 77 per 10 HPFs under ×400) *vs*. cases without LN metastases (median 64)	High CD68+iNOS+ and low CD68+ CD163+ amount correlates with increased OS rates by almost 50%	(170)

DFS, disease-free survival; DSS, disease-specific survival; CRP, C-reactive protein; HPF, high-power field; IF, immunofluorescence; IHC, immunohistochemistry; LN, lymph node; NSCLC, non-small lung cancer; TAMs, tumor-associated macrophages; OS, overall survival; RFS, recurrence-free survival; TMA, tissue microarray.

Thus, in patients with NSCLC, the expression of CD68 in tumor tissue was significantly higher in comparison with normal tissue, and high amount of CD68+ macrophages positively correlated with a higher TNM stage, peritumoral LVD, and LN metastasis (56, 165). Association between infiltration of CD68+ macrophages and EGFR-status was demonstrated in study of 105 surgically resected tumor samples (50 EGFR mutated and 55 EGFR wild-type) (171). CD68+ cells within the tumor niche exhibited more intensive infiltration in wild-type EGFR than in mutated tumors, and were related to lymph node invasion (171).

Similar to breast cancer the intratumoral localization of TAMs can have distinct role on the prognosis. IHC analysis of 99 patients with NSCLC demonstrated that the number of CD68+ macrophages in the tumor islets was positively associated with OS, whereas the number of macrophages in the tumor stroma was negatively associated with OS (172). However, specific phenotypes in tumor islets and stroma were not identified in this study, and the role of CD68+ TAM amounts in lung cancer metastasis was not clarified.

#### Subpopulations of TAMs in Lung Cancer Progression

TAM phenotype in lung cancer is characterized mostly by M2-like markers, such as CD163, CD204, and MARCO. A number of studies demonstrated that M2 macrophage phenotype positively correlates with poor survival and efficient development of metastasis in lung cancer. In order to elucidate the biological and clinical significance of M2 TAMs, a comprehensive clinical study that assessed tissue distribution of CD163+ TAMs in tumor stroma, tumor islets, and alveolar space in 160 NSCLC patients from the Japanese cohort was performed (166). Thus, high stromal and alveolar density of CD163+ TAMs significantly correlated with the C-reactive protein (CRP) level in circulation, the Ki-67 proliferation index and invasive size, tumor differentiation, lymph node metastasis and pathological stage (166). The DFS and OS were significantly lower in patients with high infiltration of stromal and alveolar CD163+ TAMs. The islet CD163+ TAMs were not associated with these parameters (166). Availability of all quantitative parameters in this study used as thresholds for TAM density in stromal and alveolar compartments merits our attention as an example of scientific transparency and clarity (166).

A study of 335 patients with stage I–IIIA NSCLC from the Danish cohort revealed the association of the density of CD163+ macrophages in tumor nests and stroma with elevated CRP level and LN metastases, but no correlation with RFS or OS was found (167). The significant accumulation of CD163+ TAMs in malignant pleural effusion of lung cancer patients closely correlated with reduced PFS (173). CD163+ macrophages were the predominant macrophage subpopulation detected in bronchoalveolar lavage fluid (BALF) from lung cancer patients (174, 175). However, no significant correlation of CD163+ macrophages in BALF with clinical and pathological parameters was found, indicating prognostic role of CD163+ TAMs in tumor tissue, but not in BALF.

In contrast to other tumor types that are considered in the present review, most pronounced prognostic significance of CD204+ macrophages in lung cancer was shown in a number of studies of Japanese cohort of patients (**Table 4**). Thus, in 297 samples obtained from patients with NSCLC, high density of CD68+ or CD204+ TAMs (assessed independently by IHC) in tumor stroma, but not in tumor islets or alveolar space, positively correlate with an advanced disease stage and histological grade, pleural invasion, node status, and wild-type EGFR gene status, and poor DFS of NSCLC patients (168). Similarly, CD204+ macrophages in the tumor stroma of 201 patients with lung adenocarcinoma positively correlated with tumor differentiation, pathologic stage, T status, nodal involvement, lymphatic permeation, vessel invasion, and pleural invasion (176). Besides, the numbers of CD204+ macrophages significantly correlated with microvessel density and the numbers of Foxp3+ lymphocytes and the expression levels of IL-10 and MCP-1 (176, 177). High levels of CD14+CD204+ cells in the pulmonary vein (PV) of patients with NSCLC were identified in cases of early recurrence and were positively related to the expression of CD204 in the tumor stroma of 207 stage I lung adenocarcinoma patients from Japanese cohort (178).

Controversial data have been obtained in a Norway study of 553 primary NSCLCs. It was found that high levels of CD204+ M2 as well as CD68+/HLA-DR+ M1 and CD68+ infiltration in stromal and intratumor compartments were independently associated with improved NSCLC-specific survival (169). HLA-DR+/CD68+ M1 TAM level significantly decreased from pathological stage I to stage III. In lymph nodes, the intratumoral level of HLA-DR+/CD68+M1 was an independent positive prognostic indicator (169). Technologically, this study differed from the previous ones by using multiplex chromogenic immunohistochemistry in tissue microarrays.

MARCO was defined as one more M2 marker of TAMs in lung cancer. Multiplex immunofluorescent staining of tumor samples from NSCLC Swedish patients demonstrated the co-localization of CD68, CD163, and MARCO (179). Co-staining of PD-L1, MARCO, and CD68 revealed MARCO+ TAMs are in direct contact with PD-L1+ tumor cells and demonstrated co-localization of MARCO and PD-L1 in TAMs (179). RNA-seq analysis of 199 tumor tissues from the same Swedish cohort showed the positive correlation of MARCO gene expression with the expression of genes associated with immunosuppressive TAMs (CD163, CD204, IL4R, CHIA, TGFB1, and IL10), genes of regulatory T-cells (FOXP3, TGFB1, IL10, EBI3, PDCD1, and CTLA4), genes of exhausted T-cells (PDCD1, CTLA4, TIGIT, BTLA, HAVCR2, and LAG3), genes of cytotoxic T-cells (CD8A, PRF1, GZMA, and GZMB) and genes of immune checkpoint molecules PD-L1, VISTA, PD-1, and CTLA4 (179). MARCO-expressing TAMs which may be considered as a specific macrophage subpopulation contributed to an immunosuppressive mechanism protecting cancer cells.

The distribution of M1 and M2 macrophages in tumor islets and tumor stroma may differ and can be associated with survival rates in NSCLC patients (170). Thus high infiltration of M1 macrophages (CD68+iNOS+) in tumor islets was associated with increased overall survival (OS) in NSCLC, while high infiltration of total M2 macrophages (CD68+CD163+) in tumor islets and stroma was associated with reduced OS in NSCLC (170).

In lung cancer TAMs have a great heterogeneity, and a number of studies demonstrated the prognostic value of TAMs expressed specific markers. For example, TAMs isolated from 96 primary lung cancer tissues displayed the elevated level of cathepsin K, COX-2, MMP-9, PDGF-B, uPA, VEGFA, and HGF (180). MMP9 and VEGF expression was significantly higher in patients with LN metastasis and lymphovascular invasion (180). Recently, using LLC-induced tumors of MafB-GFP knock-in heterozygous mice, transcription factor MafB was detected to be specifically expressed in CD204+ TAMs (181). Immunostaining analysis of human lung cancer tissue revealed that MafB is expressed in the same region and mostly in severe samples together with CD204+ and CD68+ TAMs (181). In peripheral blood collected from patients with lung carcinoma, B7-H4-expressing CD68+ macrophages were found. The level of B7-H4-expressing macrophages was significantly higher in lung cancer patients in comparison with healthy donors and was related to tumor size, lymph node metastasis, and TNM stage (182). CD68+ macrophages also expressed the protein V-set and Ig domain-containing 4 (VSIG4), a novel B7 family-related macrophage protein which has the capacity to inhibit T-cell activation; however, no correlations of VSIG4+ TAMs with patient’s outcome was found up to this date (183). Triggering receptor expressed on myeloid cells (TREM)-1, a molecule crucial for the triggering and amplification of inflammatory response was found to be expressed on TAMs in NSCLC. TREM-1+ TAMs in tumor tissue of patients with NSCLC were associated with reduced DFS and OS (184). SPP1 expressed by TAMs was indicated as an independent predictor for OS and DFS, especially for stage I NSCLC (185). TMA analysis of 159 lung cancer tissue samples demonstrated that MVD was increased in patients with positive expression of SPP1 in TAMs compared with that in the SPP1-negative group (185). IHC analysis of 213 cases of human lung adenocarcinoma specimens revealed that PD-1 is preferentially expressed by CD163+ TAMs in the tumor stroma, and these stromal PD-1+ TAMs were an independent predictor of reduced survival in lung cancer patients (57). Furthermore, PD-1+ TAMs possess a unique transcriptional profile as compared to PD-1− TAMs as was shown in mouse allografts of lung adenocarcinoma (57).

### TAMs and Lung Cancer Treatment

The primary treatment for early stage lung cancer (Stages I and II) is surgery which provides long-term survival in patients. Five-year OS after surgical resection is 60–80% for patients with stage I NSCLC and 30–50% for patients with stage II NSCLC (151). In patients with unrespectable tumors, primary radiotherapy is used. The platinum-based chemotherapy used in adjuvant regimen is beneficial for stage II NSCLC patients (151).

For advanced lung cancer (Stage IV) the treatment with platinum (cisplatin or carboplatin)-based chemotherapy in combination with taxanes (paclitaxel, docetaxel, or vinorelbine), antimetabolites (gemcitabine or pemetrexed), or vinca alkaloids (vinblastine) is recommended as a first-line therapy (151, 153).

Lung cancer cells can carry mutations in a number of proto-oncogenes including KRAS, EGFR, BRAF, PI3K, MEK, and HER2, making targeted drug to be attractive treatment strategy (152, 153). The first of the approved targeted drugs for NSCLC patients are anti-EGFR agents, tyrosine kinase inhibitor (TKI) Erlotinib (Tarceva) and gefitinib (Iressa). Gefitinib might be recommended as a first-line therapy for patients with EGFR mutations, while chemotherapy is preferred if EGFR mutation status is negative or unknown. Anti-VEGF inhibitor (Bevacizumab) is also used for the treatment of lung cancer (151). Bevacizumab in combination with first-line platinum-based chemotherapy showed significantly improved response rates, PFS, and OS compared to chemotherapy alone (153). Several clinical trials investigated therapeutic approaches that combine Immune Checkpoint Inhibitors (anti-CTLA4, anti-PD1, anti-PD-L1) and chemotherapy in NSCLC (152). However, resistance to these treatments frequently occurs that makes the development of new antitumor strategies based on immunomodulation highly relevant.

Contradicting results are available for the association between macrophage polarization and the antitumor effect of distinct drugs (*e.g.* chemotherapy, tyrosine kinase inhibitors) (**Table 7**). In patients with stage II/III NSCLC (USA cohort), treated by platinum-based NAC, density of CD68+ TAMs was higher than in untreated patients (186). In NAC treated patients higher levels of TAMs both in tumor nest and stroma were associated with better OS (186). In contrast, low total macrophage number defined by CD68 expression is an independent factor for better DFS in pN2 stage IIIA NSCLC patients receiving neoadjuvant chemotherapy (NAC) (cisplatin/docetaxel) from the Chinese cohort (187). However, high tumor islet/stromal macrophage ratio was significantly associated with longer DFS and OS (187). In a French study of 122 stage III-N2 NSCLC patients treated with cisplatin-based chemotherapy, no correlation of CD68+ TAMs with survival rates was found (188). These data indicated TAMs located in tumor nest (islets) as a favorable prognostic factor after platinum-containing chemotherapeutic treatment.

Several studies indicated the influence of chemotherapy on circulating monocytes in lung cancer. Thus, the absolute number of total CD14+ monocytes (taken before treatment) in peripheral blood of patients received adjuvant cisplatin-based chemotherapy was significantly increased in patients with progressive disease (PD) after chemotherapy in comparison to patients with partial response (PR) or stable disease (SD) (189). Percentage and absolute number of CD14+HLA-DR^−/low^ MDSCs were significantly increased in patients with PD compared with patients with PR and SD after chemotherapy (189). Besides, the low amount of CD14+HLA-DR^−/low^ cells was associated with longer PFS (189). Significant increase of IL-1beta (M1 cytokine) and significant decrease of IL-1ra (M2 cytokine) production by alveolar macrophages isolated from BALF after platinum-based chemotherapy were demonstrated in patients with small cell lung cancer from the Japanese cohort (190). It was also found that platinum-containing drug oxaliplatin induced immunogenic cell death (ICD) in LLC cells, activating dendritic cells with CD80+CD86+ phenotype and enhancing cytotoxic CD8+ T cells in LLC tumor tissues, which resulted in tumor regression in a mouse model of lung cancer (191). However, no effect of oxaliplatin on macrophages was investigated in this study (191).

Tyrosine kinase inhibitors (TKIs) were found to have an impact on the polarization of TAMs. In the study of 206 stage IIIb or IV NSCLC patients treated with EGFR-tyrosine kinase inhibitors (gefitinib or erlotinib), stromal TAMs were the predominant CD163+ TAMs (192). Among all patients as well as patients with EGFR mutation, TAM density was significantly related to poor PFS and OS (192). In contrast, in LLC-derived mouse model, Gefitinib (EGFR inhibitor) and Imatinib (tyrosine kinase inhibitor) inhibited the M2-like polarization of macrophages by reducing expression of CD206 and CD163 and M2-like genes (*Arg1, Mgl2, Ym1, Fizz1, IL-10, CDH1, CCL2*). This promotes anti-metastatic effect of Gefitinib and imatinib (193, 194). The combination of Gefitinib/simvastatin with anti-PD-L1-modified liposomes or with Vorinostat (histone deacetylase inhibitor) demonstrated better antitumor effect by repolarization of macrophages (inhibition of CD206, ARG-1 expression and activation of CD86, iNOS expression, and ROS production) and inhibition of revascularization (downregulation of VEGF, HIF-1a and CD31 expression) in lung cancer cell lines (195, 196). Vorinostat had an impact on TAM re-polarization. In mouse lung tumor tissues, the percentages of F4/80+ CD206+ cells and CD68+CD206+ cells were decreased at the 7th day after the administration of Imatinib (194).

Recent case report is available that suggested that TAMs in lung cancer can be a predictor of a positive response to anti-PD-1 antibodies (nivolumab) in patents with *EGFR*-mutated lung cancer (197). In this case report a 72-year old male patient with lung adenocarcinoma (cT1bN2M0, cStage IIIA) was harboring anEGFRexon19 deletion. The patient was subjected to right upper lobectomy after NAC. Twelve months after the surgery, recurrence of multiple brain metastases was identified, and the brain lesions were treated with *γ*-knife therapy. Thirteen months after radiosurgery, multiple lung metastases have been identified by CT. Chemotherapies, including EGFR tyrosine kinase inhibitors (TKIs), erlotinib, carboplatin plus paclitaxel, and docetaxel, were then administered consecutively. Erlotinib as second-line therapy was continued for seven months with a partial response. However, multiple lung metastatic lesions regrew. Although, the PD-L1 expression was negative, nivolumab was administered as sixth-line therapy. After seven cycles of nivolumab administration, the patient has continued treatment with nivolumab for more than two years with no evidence of tumor regrowth or serious immune-related adverse events (197). TAMs were analyzed in lung tumor by IHC, and CD68, CD206 and PD-L1 expression was detected (197). However, this study does not provide any evidence for the dynamic changes of TAM amounts or phenotypes in primary tumor and metastatic sites and also during different chemotherapy approaches. The presence of TAMs does not explain their role in the tumor spread and response to various chemotherapy approaches. In lung cancer patients of Italian cohort, CD163+CD33+PD-L1+ macrophages with epithelioid morphology (alveolar macrophage-like) defined by the authors as “complete immunophenotype,” were detected in all patients with hyperprogression. The authors suggested that CD163+CD33+PD-L1+ TAMs are statistically significantly associated with hyperprogression compared to patients without hyperprogression (198). However, it is hard to understand whether CD163+CD33+PD-L1+ TAMs can be also found in small amounts in patients without hyperprogression. These reports show that our knowledge about the role of TAMs in response to various types of chemotherapy as well as to immunotherapy in patients is strictly limited. They highlight the urgent need to intensify investigations in this field.

In summary, several lines of evidence show that TAMs can improve the response of lung cancer patients to chemotherapy, in particular their higher amount in tumor nest in case of platinum-based chemotherapy. Increased amount of circulating monocyte that can be recruited to tumor mass and differentiate into TAMs is rather a negative factor for the patient response to cisplatin-based chemotherapy. TAMs correlated with poor response to EGFR-tyrosine kinase inhibitor Gefitinib, while in mouse models Gefitinib induced re-polarization of TAMs to antitumor phenotype. The role of TAM in immunotherapy of lung cancer needs careful analysis. The mechanism of TAM interaction with of anti-lung cancer treatments has to be identified in order to develop new immunomodulating approaches.

## TAMs and Ovarian Cancer

Ovarian cancer (OC) is the most lethal gynecological cancer (199). Around 300 thousand new cases of ovarian carcinoma are diagnosed worldwide in 2018, with around 184 thousand deaths (34). The origin of more than 90% of malignant ovarian tumors is epithelial. Epithelial OC is a heterogeneous disease with histological subtypes that differ by cellular origin, pathogenesis, and prognosis (199, 200). Epithelial OC consists of five main histotypes: high-grade serous (HGSOC; 62%), endometrioid (ENOC; 20%), clear cell (CCOC; 8%), mucinous (MOC; 5%), and low-grade serous (LGSOC; 5%) (199, 200). High-grade serous ovarian carcinoma (HGSOC) is often diagnosed at the late stages and exhibits the highest aggressiveness and mortality (201).

The biological behavior of ovarian carcinoma differs from other tumors by the pattern of hematogenous metastasis through transcoelomic dissemination of tumor cells *via* the peritoneal fluid (202, 203). In ascite, cancer cells detached from the primary tumor obtain EMT phenotype, form multicellular spheroids and attach preferentially on the abdominal peritoneum or omentum through a passive mechanism, carried by the physiological movement of peritoneal fluid (203). Floating spheroids form a continuously repopulated chemoresistant niche, that leads to the high mortality of patients with cure rate of only 30% (203).

There are no effective criteria to diagnose OC at early stages, and screening tests for ovarian cancer are limited in sensitivity. Therefore, up to 70% of cases are detected at the advanced stages (204). The five-year survival of patients with disseminated tumors is only about 25% at the stage III and not more than 5% at the stage IV stage (according to International Federation of Gynaecologists and Obstetricians (FIGO) (205). Despite a good response of disease to the first line of standard platinum/taxane-based chemotherapy (cisplatin or carboplatin and paclitaxel or docetaxel), development of recurrence associated with multidrug resistance is detected within a short period in 70% patients (206). Moreover, it was shown that these chemotherapeutic agents, as well as anthracyclines and cyclophosphamide, can contribute to metastasis (206). It is not excluded that such pro-metastatic effect can be due to the detrimental effects of the therapeutic agents on the components of TME, including TAMs. However, the effects of chemotherapeutic agents on TAMs in ovarian cancer remain to be investigated. So it is necessary to develop more effective approach to cure the patients who have acquired drug resistance during standard chemotherapy, and this approach has to include programming of intratumoral immunity.

### TAMs in Ovarian Tumors and Metastasis

By analysis of the role of macrophages in OC progression both TAMs infiltrating tumor mass and TAMs intimately interacting with cancer cells in ascitic fluid should be taken into account.

The total number of TAMs as well as specific subpopulations in the tumor mass was examined in the patient cohorts from a broad spectrum of countries, including UK, Italy, Canada, China, Korea. The correlation of TAMs with clinical-pathological parameters (TNM stage, histotypes, lymph node metastasis, hematogenious metastasis) and survival rates was analyzed. Similar to breast cancer, a number of studies demonstrated positive correlation of TAMs with poor prognosis in OC. However, in contrast to breast cancer, CD68 was not frequently used as TAM marker to evaluate TAM levels (**Table 5**, **Figure 1**). Thus, in the study of 332 patients with high-grade serous ovarian carcinoma (HGSOC) from the UK cohort, stromal CD68 expression was found to be positively associated with survival rates (207). In 112 ovarian cancer patients from the Chinese cohort, intratumoral CD68+ TAM density significantly increased with increasing cancer stage and grade, however, displayed no prognostic significance in both the Kaplan–Meier survival and multivariate Cox regression analyses (208).

**Table 5:** Representative studies demonstrating the association of TAMs with tumor progression parameters in ovarian cancer.

Cohort of patients	Method of detection	TAM correlation with tumor growth and stage	TAM correlation with lymphatic and hematogenous metastasis	TAM correlation with survival	Reference
332 HGSOC patients (UK)	IHC in TMA (digital imaging scanning)	Not studied	Not studied	High amount of stromal CD68+ TAMs is associated with increased OS rate by 15%	(207)
112 ovarian cancer patients (China)	IHC (manually)	1.6–2.0-fold increase of CD68+ and CD163+ TAM densities is found in tumors with grade G3 *vs*. grade G1. Decrease in M1/M2 TAM ratio is observed from stage I (1.4 ± 0.5 cells/mm^2^) to stage IV (1.0 ± 0.5)	Not studied	Increase of overall M1/M2 ratio above the mean 1.731 is associated with increased 5-year OS by 19.7%	(208)
110 EOC patients (China)	IHC (manually)	1.7-fold increase of CD163+ TAM amount is found in tumors with grade 2–3 (median = 79 cells) compared to grade 1 (median = 47 cells)	Not studied	Increase of CD163+ TAM amount above the median (76 cells per ×400 HPF) correlates with decreased PFS rate by 25.7% and OS rate by 26.9%	(209)
140 ovarian cancer patients (Italy)	Flow cytometry	Not studied	Not studied	High M1/M2 ratio (defined as 1.4) is associated with prolonged OS by 16 months, and PFS – by 15 months compared to low M1/M2 ratio (< 1.4).	(210)
199 HGSOC patients (Canada)	IHC of TMA (digital imaging scanning)	Not studied	Not studied	Increased CD206+/CD68+ ratio correlates with decreased OS and PFS rates by 40%	(58)

DFS, disease-specific survival; DSS, disease-free survival; HPF, high-power field; IF, immunofluorescence; HGSOC, high-grade serous ovarian cancer; IHC, immunohistochemistry; LN, lymph node; TAMs, tumor-associated macrophages; OS, overall survival; RFS, recurrence-free survival; TMA, tissue microarray.

#### Subpopulations of TAMs in Ovarian Cancer Progression

The association of macrophage polarization with survival of ovarian cancer patients was demonstrated in numerous studies that used M1 and M2 markers for the phenotyping of TAMs or M1/M2 ratio (**Table 5**). Meta-analysis of nine studies (eight from Chinese cohorts and one from USA cohort), including 794 patients, revealed that higher M1(iNOS+ or HLA-DR+)/M2(CD163+) ratio, but not just CD68 or CD163 expression in tumor tissues, was associated with a favorable OS (211). Besides, elevated M1/M2 ratio predicted better PFS of ovarian cancer (211). In contrast, worse PFS was associated with high density of CD163+ TAMs and higher ratio of CD163/CD68. High density of CD163+ and CD68+ TAMs was observed in OC with advanced TNM stage (211). IHC analysis of 110 Chinese patients with stages III–IV epithelial ovarian cancer revealed that PFS and OS rates were higher in the low-CD163 expression group than in the high-CD163 expression group (209). CD68 expression did not show significant differences, while the high CD163/CD68 ratio was a negative predictor for PFS and OS (209). In the study of the Chinese cohort that enrolled 112 OC patients, the M1 (HLA-DR+)/M2(CD163+) ratio also positively correlated with 5-year survival rates (208). Decrease in M1/M2 ratio was observed in cancer specimens from Stage I through Stage IV. At the same time, high number of CD163+ TAMs was associated with increasing cancer stage and the size of the residual site (208). In patients from the Italian cohort a positive relationship between the M1(CD14+CD80+)/M2(CD14+CD163+) ratio and OS and PFS was found in patients with HGSOC and patients with other histotypes or ovarian metastases (210). High serum levels of CD163 in Korean patients with EOC were associated with advanced stage and with short DFS and OS (212). The density of CD206+ macrophages was not prognostic, but a higher ratio of CD206+/CD68+ cells was strongly associated with worse PFS and poorer OS that was found by IHC analysis in a cohort of 199 HGSOC patients from the Canadian cohort (58).

There is evidence about the differences in TAM clinical value between different histological types of ovarian cancer. Thus, the numbers of CD68+ macrophages, as well as the numbers of macrophages positive for M2 markers (CD163 and CD204) in borderline and malignant tumors were significantly higher in both serous and mucinous ovarian tumors than in benign tumors (213). As for serous carcinoma, total CD68+ macrophage infiltration together with CD163 expression was significantly increased in high-grade serous ovarian cancer (HGSOC) compared to low-grade serous ovarian cancer (LGSOC) (214). At the same time LGSOC had significantly lower microvessel density assessed by CD31 and lower MMP9 expression (214).

Other studies found the associations of macrophages expressed different specific factors with clinical and pathological parameters in ovarian cancer (**Table 1**). In peripheral blood of 51 patients with pathologically diagnosed ovarian cancer the proportion of PD-L1+ CD68+ cell among CD68+ cells and the intensity of PD-L1 staining on CD68+ cell were significantly higher in the ovarian cancer group in comparison with the healthy group (215). Besides, these parameters were increased at the late stage cancer (stages III–IV) compared to early stage cancer (stage I–II) (215). IHC and immunofluorescent analysis of tumor samples from 102 OC patients of Chinese cohort showed that reduced ratio of M1(HLA-DR+ or iNOS+)/M2(CD163+ or VEGF+) TAMs and the increased densities of COX-2+ TAMs were the predictors of poor prognosis (216).

B7-H4 (the member of the B7 family of T cell costimulatory molecules, is a negative regulator of T cell responses) was found to be expressed by TAMs in ovarian cancer. Primary ovarian tumor cells express intracellular B7-H4, whereas TAMs have surface B7-H4 expression (217). B7-H4+ tumor macrophages expressed higher levels of CD86 than B7-H4-tumor macrophages, but the expression of other molecules responsible for T cell activation (HLA-DR, HLA-ABC, CD40, and CD80) did not differ. *In vitro* and *in vivo*, B7-H4+ TAMs, but not cancer cells, suppressed T cell immunity. Blocking B7-H4, but not arginase, inducible nitric oxide synthase or B7-H1 restored the T cell stimulating capacity of the macrophages and contributed to tumor regression *in vivo* (217).

Gene chip analysis showed that human TAMs express significantly higher levels of insulin-like growth factor 1 (IGF1) than undifferentiated M0 myeloid cells (218). *In vitro* TAMs may increase the proliferation and migration of ID8 mouse EOC cells by upregulation of IGF1. Blockade of the IGF1 pathway in ID8 cells with an IGF1 neutralizing antibody effectively inhibited the proliferation and migration of ID8 cancer cells (218). Using histological data obtained from 395 EOC patients, it was found that CD163+ TAM infiltration correlates with higher expression of ZEB1 that drives EMT in ovarian cancer cells (219). ZEB1 expression was identified in TAMs, and ZEB1+TAMs correlated with poorer survival and higher expression of CCR2 and MMP9 in patients with EOC. Mouse TAMs that expressed *Zeb1* were prone to the polarization toward an F4/80^low^ pro-tumor phenotype and accelerated tumor growth (219). IHC study of 108 samples from patients with EOC demonstrated that CD68+ TAM infiltration and high-mobility group box protein 1 (HMGB1) expression closely correlated with lymph node metastasis and with poor OS (220). *In vitro*, TAMs isolated from ascites of EOC patients and HMGB1 facilitated lymphangiogenesis by inducing LEC proliferation, migration, and capillary-like tube formation (220).

#### Ascitic TAMs in Metastasis of Ovarian Cancer

In ovarian cancer TAMs have a clinical significance not only by infiltrating tumor mass but also by the interacting closely with cancer cells in ascites. Ascite, which is a hallmark of OC, contains a large number of components of unique peritoneal TME, including tumor spheroids and immune cells, such as TAMs and T cells (201, 202). Experimental mouse models have demonstrated that TAMs constitute a major cell fraction in ascites that support the survival of cancer cells and promote cancer progression, chemoresistance, and immunosuppression (202, 204, 221–223).

Interestingly, TAMs were found to maintain transcoelomic metastasis by tumor spheroids (221). As was shown, in tumor spheroids isolated from 128 patients (USA cohort) with advanced stage OC, higher amounts of CD68+ macrophages were found in poorly differentiated OC compared with more-differentiated OCs, and their amount correlated with lymphovascular invasion (LVI) and ascite volume. High number of CD68+ macrophages in these spheroids was significantly associated with lower 5-year OS of patients (221). In a mouse model of ovarian cancer, EGF, secreted by TAMs, promoted early transcoelomic metastasis. Immunostaining of mouse tumor spheroids isolated from ascite, confirmed that EGF was specifically detected in TAMs that were surrounded by EGFR+ tumor cells. Pharmacological blockade of EGFR or neutralizing antibody for ICAM-1 in TAMs blunted spheroid formation and ovarian cancer progression in mouse models. These findings suggest that TAMs play an essential role in spheroid formation during the process of transcoelomic metastasis of OC (221).

The possibility to isolate high amount of pure macrophages from the ascitic fluid enables high throughput analysis of their transcriptome and proteome. The transcriptomic and proteomic analysis of TAMs in ascites of OC patients was performed in detail by the group of R. Muller (224–226). Transcriptomic analysis (RNA-seq) of TAMs isolated from 18 ascites of ovarian cancer patients (Germany cohort, serouse, and clear cell carcinoma) revealed two signatures of expressing genes: signature A, characterized by the hyperexpression of pro-tumor markers (CD163, PCOLCE2, IL6) related to ECM remodeling and signature B with low expression of pro-tumorigenic and immunosuppressive markers and an upregulation of genes linked to interferon signaling (225). It was shown that subgroup A of TAMs correlated with a short OS, while subgroup B linked to a favorable clinical outcome in OC patients (225).

RNA-seq analysis also revealed that CD163+ or CD206+ TAMs isolated from the ascites of HGSOC patients (Germany cohort) have elevated expression of protumorigenic growth factors and cytokines, *e.g.* CCL18, KITLG, SEMA6B, S100B, and VEGFB and downregulated tumor suppressive mediators, *e.g.* CXCL10, CXCL11, IL15, TNFSF10, and TNFSF14 (226). The increased expression of proteins involved in ECM remodeling (*ADAMTS2, CTSB, FBLN5*) and complement factors (*C1QC* and *CR1L*) was also found in CD163 or CD206-expressing TAMs. TAMs from ascites also produce CCL5, CXCL8, IL1RN, CCL18, CXCL2, CXCL3, acting as a chemokines for the monocyte/macrophage recruitment (226). The gene expression of IL10, TGFbeta1, S100A8, S100A9, and IL10RA was upregulated in TAMs compared to tumor cells isolated from the ascites of OC patients (227).

Surprisingly, flow cytometry analysis identified that neither CD163 nor CD206 distinguishes TAMs (from ascite of 79 OC patients) from resident peritoneal macrophages (pMPHs) (from 11 patients undergoing hysterectomy for non-malignant diseases (ovarian cyst, uterine myomatosis, endometriosis) (224). RNA-seq data confirmed that TAMs closely resemble pMPHs (224). Both TAMs and pMPHs expressed a number of macrophage markers, including phagocytosis-associated receptor genes (*CD36, MSR1, SCAR* family genes,*TIMD4, CD163*), *FCGR* genes, complement receptor genes (*CD93/C1Q-R1, C3AR, CR1, C5AR1*), and polarization marker genes (*IL10*). However, upregulation of ECM remodeling genes (*COL* family genes, *LUM, PCOLCE2*) was selectively observed only in ovarian cancer TAMs (224). The limitation of this study may be due to the comparison of TAMs from OC patients and pMPHs from the patients with non-malignant diseases, but not pMPHs from healthy donors.

### TAMs and Ovarian Cancer Treatment

Patients with stage I ovarian cancer undergo surgery. Treatment of stages II–IV of epithelial OC includes complete surgical resection, followed by platinum-based chemotherapy. Another option is NACT, interval cytoreductive surgery, followed by adjuvant platinum/taxane chemotherapy (228, 229). Platinum and taxane combination as chemotherapeutic treatment showed improved survival in early stage OC of high-grade lesions (216). In the past 2 years the interest to the problem of the interaction of chemotherapy and TAMs in OC has been increased and some novel data were accumulated.

Cisplatin is a most frequently used conventional drug in ovarian cancer patient (228). *In vitro*, cisplatin stimulated human macrophage-like THP-1 to become classically activated (CAMs) and to produce CCL20, chemokine ligand 20 (macrophage inflammatory protein-3 (MIP3A), that activates CCR6 on ovarian cancer cells, promoting EMT and migration (230). Cisplatin has only limited effect on the polarization of CAMs, by increasing IL-1*β* expression, but not affecting other typical M1 (TNFα, iNOS) and M2 (IL-10, ARG-1, CCL18) polarization markers. The specific blockade of CCL20 on CAMs as well as inactivation CCR6 on tumor cells by siRNA diminished cisplatin-induced cancer cell migration. Thus, a novel pro-migration mechanism driven by the crosstalk between cisplatin and CAMs, allow to consider the CCL20-CCR6 axis for therapeutic targeting to reduce chemotherapy-induced metastasis in advanced stage ovarian cancer (230). *In vitro* in co-culture of THP-1 macrophages and A2780 cancer cells, cisplatin downregulated expression of CD274, IL-6 and HLA-DRA without inducing M2-type markers in M1-type macrophages, while doxorubicin caused the decrease in HLA-DRA and increase in CD206 (231). In M2 macrophages, downregulation of CD163 and IL10 under doxorubicin treatment was observed (231).

Recently molecular profiling of more than 500 genes was performed, and 22 immune subsets were estimated with computational analysis CIBERSORT in 13 studies that enrolled 2,218 patients with HGSOC, who underwent platinum-based chemotherapy. As was found, a high fraction of M1 and M0 macrophages was associated with favorable OS, whereas the M2 macrophages conferred worse OS that was found by CIBERSORT approach (232). In the study from Netherlands, which enrolled 69 peritoneal samples from patients with HGSOC who underwent NAC, an increase in CD3+ cells in peritoneal metastases of HGSOC was observed and an increase of CD3+ and CD8+ cells was associated with improved PFS and OS; however, no correlation between TAM number and outcome was found after NAC (233). Patients with HGSOC from the Italian cohort treated with adjuvant cisplatin-based chemotherapy (cisplatin/carboplatin + Taxol + bevacizumab) had a significantly higher M1/M2 ratio in platinum-sensitive tumors compared to platinum-resistant tumors (210) (**Table 7**).

Paclitaxel is the antitumor agent which enables the rearrangement of microtubules resulting in cell cycle arrest in tumor cells (2). Paclitaxel can also program the immune system for tumor inhibition. The microarray analysis of tumors derived from OC patients undergoing paclitaxel chemotherapy revealed that paclitaxel exposure results in the increase in genes linked to the M1 macrophage activation profile (IFNg-stimulated macrophages) in comparison with gene profile before treatment (234). *In vitro* TAM phenotype skewed to M1-like one mediated by TLR4 innate immunity receptor. This study endows new evidence that the antitumor effect of paclitaxel occurs in part *via* reactivation of the immune response against cancer, with repolarization of TAMs toward the M1-like antitumor phenotype (234).

*In vitro* and *in vivo* treatment with paclitaxel and carboplatin increased MCP-1 expression in ovarian cancer cells that is known to be responsible for inducing macrophage migration (235). Chemotherapy with paclitaxel or carboplatin may generate debris in ID8 ovarian cancer cells which triggers macrophage production of the proinflammatory cytokines TNF-α, MIP-2/CXCL2, MIP-1*β*/CCL4, CCL2/MCP-1, as well as sICAM-1/CD54 and G-CSF (236). Cytokine storm induced by debris-stimulated macrophages was prevented by the dual cyclooxygenase-2 (COX-2) and soluble epoxide hydrolase (sEH) inhibitor PTUPB. Indeed it may be an approach to suppress debris-stimulated ovarian tumor growth by preventing the therapy-induced surge of cytokines and lipid mediators (236). Hyaluronic acid-based nanoparticles encapsulating miR-125b (HA-PEI-miR-125b) specifically target TAMs in the peritoneal cavity of a syngeneic ID8-VEGF ovarian cancer mouse model and repolarize macrophages to an immune-activating phenotype (increased CD80 and iNOS and reduced CD206 and ARG1 expression) (237). Intraperitoneal administration of paclitaxel in combination with HA-PEI-miR-125b nanoparticles enhanced the antitumor efficacy of paclitaxel mediating by the significant reduction in the ascite fluid and peritoneal VEGF levels (237). Docetaxel treatment increased the infiltration of macrophages in ID8 tumor-bearing mice. Docetaxel in combination with BLZ945 (CSF-1R inhibitor) treatment significantly inhibited tumor growth, reduced the abundance of TAMs, increased CD8+ T cell infiltration and prevented lung metastasis in a mouse epithelial ovarian cancer (238). Imminofluorescence/confocal analysis of 24 patients with OC (Belgium cohort) who underwent platinum-based neoadjuvant chemotherapy (carboplatin and paclitaxel) revealed an increase in vessel width, TAMs, and M2-like macrophages after NAC (239). Blood vessel width was correlated with M2 presence. The additional use of bevacizumab (anti-VEGF therapy) resulted in more pronounced increase in the number of TAMs and M2 macrophages compared to paclitaxel–carboplatin alone (239).

A phase 1/2 study of 18 patients who had platinum-resistant ovarian cancer (the Netherlands) showed that gemcitabine reduced myeloid-derived suppressor cells and increased immune-supportive M1 macrophages (240). Combination of gemcitabine and Pegintron (IFN-alpha) stimulated higher portions of circulating CD4+ and CD8+ T-cells but not regulatory T-cells. All patients vaccinated with p53 synthetic long peptide (SLP) vaccine showed strong specific T-cell responses. Combination of gemcitabine, the immune modulator Pegintron and therapeutic peptide vaccination is a new approach of combined chemo-immunotherapeutic regimens to treat ovarian cancer that has anti-cancer programming effect on innate and adaptive immune systems (240).

In summary, published data about the interaction of TAMs with anti-ovarian cancer treatment are highly diverse. Most of the results were generated in animal models or *in vitro*, while data from clinical studies is strictly limited. *In vitro* and animal studies demonstrated opposite effects of treatment on TAMs that depend on both experimental models and chemotherapeutic agent with different mechanisms of action. For example, cisplatin, which is a DNA intercalating agent, supported tumor-promoting functions of TAMs, while paclitaxel, affecting microtubules, induced pro-inflammatory program in TAMs. Mouse pre-clinical models and clinical trials provided promising data for the combination of chemotherapy and TAM-blocking agents that opens the perspectives for using integrated approachs in the treatment of ovarian cancer.

## TAMs and Prostate Cancer

Prostate cancer (PC) represents the second most frequent malignancy in men with an estimated over 1.5 million new cases diagnosed annually worldwide and ranks as the fifth leading cause of cancer-associated mortality globally (241). The incidence and mortality rates of PC are trending upwards due to population aging and urbanization, thereby having a significant social and financial burden on global healthcare system (242).

PC belongs to hormonally driven malignancy, whose primary progression relies on functional activity of androgen receptors (243). Accordingly, three stages in prostate carcinogenesis are distinguished: precancerous intraepithelial neoplasia, androgen-dependent, and followed by aggressive androgen-independent PC (244). Adenocarcinoma is the most common prostatic tumor, whereas other histological subtypes such as urothelial, small cell, squamous cell, and basal cell carcinomas are diagnosed quite rarely (245). The major routes for PC progression include extracapsular extension and spread to pelvic lymph nodes, as well as metastasis to lungs and bones (246). Furthermore, given the abundant innervation of prostate peripheral zone, primary tumors arising in this area tend to escape the organ through perineural invasion (247).

Routine screening of PC involves an evaluation of serum levels of prostate specific antigen (PSA), a serine protease produced by prostate epithelium, while the gold standard for diagnosis confirmation is prostate biopsy analysis (248). Apart from the TNM staging system, Gleason score is used to characterize the PC metastatic potential on the basis of differentiation patterns. Thus, high-grade PC (Gleason score over 7) has higher risk of metastasis as compared to less aggressive primary tumors with Gleason score below 6 (249).

Given the hormone dependent nature of PC, androgen deprivation therapy (ADT) has been regarded as a standard treatment approach for patients with PC (250). Despite the initial efficacy and improvement in OS rates, prolonged hormonal treatment is eventually associated with the emergence of aggressive castration-resistant prostate cancer (CRPC) associated with high mortality and poor patient outcomes (251). Current evidence suggests that inflammatory microenvironment, especially TAMs, is involved in the onset of prostate carcinogenesis and acts as an essential modulator of further malignant progression, metastasis, and overall therapeutic response (252).

### TAMs in Prostate Tumors and Metastasis

In human prostate cancer, the inflammatory component of local TME is considered as an essential modulator of malignant progression and determinant of the overall therapeutic response (253). To date, a number of investigations have focused on the patterns of macrophage infiltration in prostate cancer specimens in attempts to validate its clinical and pathological significance (254) (**Table 6**). The primary analysis of TAMs in 85 prostate carcinomas (Sweden, 2000) demonstrated significant increase of the cell profile area and volume density of CD68+ macrophages in cases with higher Gleason score (260). A positive correlation was also found between the size of individual macrophage and angiogenesis measured as the number of von Willebrand factor-positive microvessels in the most vascularized area (260). In the same cohort, increased density and cell profile area of CD68+ TAMs were recognized as predictors of shorter cancer-specific survival (CSS) (260). Next study of a cohort of 81 prostate cancer patients from USA cohort revealed an increase of macrophage density in tumor versus adjacent benign tissue (255). Interestingly, a negative association between the amount of CD68+ TAM infiltrate in total tumor tissue and TNM clinical stage was found, while TAM density within cancer cell area positively correlated with Gleason score (255). Such contradicting results may reflect the heterogeneous distribution of TAMs in the tissue samples and highlights the importance of the compartment-specific macrophages in prostate tumorigenesis. High levels of CD68 in biopsy specimens of 859 patients from the USA cohort with benign prostatic hyperplasia were associated with increased risk for overall clinical progression (261). Several independent investigations confirmed high expression of CD68 in advanced prostate cancer. Thus, IHC study of 131 Japanese prostate cancer patients detected abundant CD68+ macrophage infiltration in tumor mass in patients with higher serum prostate-specific antigen (PSA) and Gleason score (59). The relapse-free survival rates in the same cohort were significantly lower in patients with greater TAM counts (59). Appropriate reporting of methodology, quantitative assessment and statistical analysis in this study could be necessary to ensure the quality of data interpretation in accordance with scientific rigor (59). Tissue microarray (TMA) containing 332 radical prostatectomy specimens (USA cohort) revealed greater abundance of CD68+ cells in malignant areas in comparison to benign tissues, as well as increase in mean TAM numbers in Gleason grade 4 *versus* grade 3 (262). IHC analysis of 100 specimens of prostate adenocarcinoma of the Turkish cohort demonstrated positive correlation between the density of CD68+ TAM infiltration and such clinical–pathological parameters as tumor stage, Gleason score, extracapsular extension, perineural invasion, and positive surgical margins (256). Furthermore, a study involving 93 prostate cancer patients from the Italian cohort identified that high expression of CD68 in primary tumor identified by IHC was an independent predictor of biochemical recurrence (defined as elevation of PSA level) after radical prostatectomy (263). Increased CD68+ macrophage count was observed in metastases from the lymph nodes, liver, bladder, rectum, and seminal vesicles in comparison to the corresponding primary tumors collected from 59 prostate cancer patients from the Norway cohort (264). Recent study of representative TMA collected from over 400 patient cohort from Germany confirmed the increase of CD68+ cell numbers in prostate cancers with Gleason score over 8 (265). Microarray analysis of 9,393 prostate cancer samples demonstrated that elevated expression signature of TAMs is strongly associated with worse distant metastasis-free survival (266). Thus, a number of studies indicated that higher CD68+ macrophage abundance in tumor tissue reflects aggressive tumor behavior and unfavorable patient outcomes in prostate cancer.

**Table 6:** Representative studies demonstrating the association of TAMs with tumor progression parameters in prostate cancer.

Cohort of patients	Method of detection	TAM correlation with tumor growth and stage	TAM correlation with lymphatic and hematogenous metastasis	TAM correlation with survival	Reference
81 prostate cancer patients (USA)	IHC (manually)	1.94-fold increase of stromal CD68+ TAM amount is found in tumors with T1a–T2a stages (mean = 228.5) *vs*. T3a stage (mean = 118.0). fivefold increase of CD68+ TAM amount is found in cancer area with Gleason score 8–10 (mean = 138.0) *vs*. Gleason score 4–6 (mean = 27.6)	Decrease of mean CD68+ TAM amount in primary cancer by 48% (from 59.3 to 30.7 cells at ×400) is associated with LN metastases	Increase of CD68+ TAM amount above the mean (185.8) is associated with increased RFS rate by 44%	(255)
131 prostate cancer patients (Japan)	IHC (not specified)	1.6-fold increase of CD68+ TAM amount is found in tumors of stage T ≥ 3 (mean = 40.54) *vs*. T ≤ 2 stage (mean = 25.26). 1.87-fold increase of CD68+ TAM amount is found in cases with Gleason score≥8 (mean = 44.94) *vs*. Gleason ≤ 6 (mean = 24.03).	Not studied	High amount of CD68+ TAMs (≥22 per ×400 HPF) correlates with decreased RFS rate by 75%	(59)
100 prostate adenocarcinoma patients (Turkey)	IHC (manually)	Increase of CD68+ TAM amount (≥15 cells under ×400, defined as score 3) is indicative for Gleason score ≥8 and stage III	High amount of CD68+ TAMs (≥15 cells under ×400) correlates with extracapsular extension and perineural invasion	Not significant	(256)
93 prostate cancer patients (Italy)	IHC (manually)	fourfold increase of mean amount of CD163+ TAMs *vs*. CD68+ TAMs is associated with Gleason score ≥ 7	Not studied	Patients with tumors of high CD163+ TAM amount show reduced biochemical RFS rates by 16% compared to those with high CD68+ TAM amount	(257)
234 prostate cancer patients (Sweden)	IHC (digital imaging scanning)	1.7-fold increase of CD163+ TAM amount is found in tumors with Gleason score ≥ 8 (mean = 100.0) *vs*. Gleason < 6 (mean = 60.1)	1.3-fold increase of mean CD163+ TAM amount (from 74.8 to 99.9) in primary cancer is associated with presence of bones metastases	Increase of CD163+ TAM amount (above 99) correlates with reduced DSS	(258)
135 prostate cancer patients (Japan)	IHC in TMA	Low amount of CD204+ TAMs (<24 cells per 0.06175 mm^2^) is associated with high PSA level (>20 ng/ml) 1.6-fold decrease of CD204+ TAM amount in tumors with Gleason score ≥ 8 (mean = 19.17) *vs*. Gleason ≤ 6 (mean = 30.2)	Not studied	Low amount of CD204+ TAMs (<24 cells per 0.06175 mm^2^) is associated with decreased RFS rate by 25%	(259)

DFS, disease-free survival; DSS, disease-specific survival; HPF, high-power field; IF, immunofluorescence; IHC, immunohistochemistry; LN, lymph node; TAM, tumor-associated macrophages; OS, overall survival; RFS, recurrence-free survival; TMA, tissue microarray.

#### Subpopulations of TAMs in Prostate Cancer Progression

Not only total macrophage amount but also specific macrophage subtypes were found to be correlated with clinical and pathological characteristics of prostate cancer patients (**Table 6**). IHC analysis of tissue specimens derived from 93 Italian prostate cancer patients has identified that high amount of CD163+ TAMs was associated with extracapsular extension (Gleason score > 7) and worse biochemical recurrence-free survival rates (257). Increased infiltration of CD163+ cells correlated with higher Gleason score and incidence of metastasis, as well as lower rates of CSS in a cohort of 234 Swedish prostate cancer patients (258). These findings were further confirmed in a study involving 592 patients with diagnosed prostate cancer from the Swedish cohort demonstrating greater CD163+ macrophage infiltration in aggressive tumors with Gleason scores ranging from 8 to 10 (267). The risk of death from prostate cancer in the same cohort was almost twofold higher in patients with high amount of CD163+ TAMs *versus* those with lower numbers (267). Positive correlation between the number of CD206+ macrophages and Gleason scores was found in Chinese cohort of 42 prostate adenocarcinoma patients (268). TMA of 192 prostate cancer samples from the USA cohort revealed greater amount of CD206+ TAMs in primary adenocarcinoma and lymphatic metastases in comparison to benign prostate tissues (269). IHC analysis of 373 prostate biopsy samples (Japanese cohort) demonstrated significantly lower numbers CD204+ TAMs in cases with prostate cancer in comparison to benign specimens (270). Negative correlation between the density of CD204+ TAMs and the clinical T stage was confirmed in the retrospective study of 135 PC patients from the Japanese cohort (259). Inverse association was demonstrated between the expression of MSR-A in primary tumors and the presence of lymph node metastases in the USA cohort of 90 prostate cancer patients (271). YKL-40 is an emerging TAM biomarker that is produced by both macrophages and cancer cells and enhances inflammation in TME (272). YKL-40 is also a strong inducer of tumor angiogenesis (273). In macrophages, YKL-40 is induced by IFN*γ* and can be considered as M1 biomarker (14, 16). Significantly higher concentrations of YKL-40 were detected in the serum of 153 patients (from Denmark) with metastatic prostate cancer compared to healthy donors (274). Accordingly, elevated plasma YKL-40 levels at the time of diagnosis were predictive of shorter OS rates in the same cohort of patients (274).

### TAMs and Prostate Cancer Treatment

To date, androgen deprivation therapy (ADT) is accepted as a standard treatment approach for patients with advanced prostate cancer (250). Despite initial efficacy and improvement in the OS, prolonged hormonal treatment is eventually associated with aggressive castration-resistant prostate cancer (CRPC) (251). Multiple lines of evidence indicate crucial role of TAMs in therapeutic response and in post-treatment recurrence of prostate cancer (275). In comparison with tumor tissues from hormone-naïve prostate cancer patients, CRPC samples displayed higher number of CD68+ macrophages expressing cathepsin S enzyme known to be involved in angiogenesis and remodeling of extracellular matrix (276). IHC analysis of 75 prostate cancer specimens (Canadian cohort) was performed in two groups of patients—patients pre-treated with Cyproterone (antiandrogen agent) or Leuprolide (gonadotropin-releasing hormone analogue) in combination with Flutamide (nonsteroidal antiandrogen) before radical prostatectomy and patients who underwent surgery only. Increase in the amount of CD68+ TAMs within tumor tissues of pre-treated patients compared to the untreated group was demonstrated (277). Increased CD68+ and CD163+ macrophage infiltration was found in a cohort of 60 Chinese prostate cancer patients receiving preoperative Bicalutamide-based ADT (278). TMA analysis was performed for retrospective cohort of 366 prostatectomized patients (Canada) divided into two groups—hormone ablation-treated patients (luteinizing hormone-releasing hormone-agonists and/or antiandrogen prior to surgery) and hormone-naïve patients. This analysis confirmed significantly higher amount of CD163+ TAMs in treated group of patients in comparison with hormone-naïve patients (279). Mouse model of prostate cancer further confirmed dramatic recruitment of TAMs in response to ADT. Substantial overexpression of VEGF-A, MMP-9, and ARG1 was found in tumors of castrated animals treated by ADT (279). Also, concentrations of CSF1, major macrophage differentiation, and chemotactic factor, were enhanced in the serum of animals in response to ADT treatment (279). In parallel, co-culture of Myc-CaP prostate cancer cells and RAW264.7 macrophages treated with antiandrogen Enzalutamide resulted in significant increase in the expression of M2 markers—VEGF-A, MMP- 9, ARG1, IL-10, and CSF1 (279). Importantly, higher levels of CD163+ macrophages were detected in the prostate cancer sections (Chinese cohort) resected after preoperative ADT in comparison to the corresponding tissues collected before therapy (280). IHC study of 126 prostate cancer patients (Italian cohort) using pelvic lymph node metastases samples obtained from those patients who received neoadjuvant hormonal treatment flutamide combined with Leuprolide acetate before radical prostatectomy was performed (281). Double IHC revealed the co-localization of CD68+ TAMs and TARC/CCL17 (thymus- and activation-regulated chemokine), chemokine regulated Treg function, in treated patients in contrast to the untreated group (281).

Clinical trial on 17 patients (USA) with Gleason score 7–10 prostate cancer, treated with anti-PD-1 therapy, revealed significant upregulation of inhibitory molecules PD-L1 and VISTA on CD68+ TAMs in tumor after treatment in comparison with baseline tumor (10-fold and fourfold increase in expression, respectively) (282). The authors suggested that VISTA expression is a compensatory pathway limiting efficiency of ipilimumab therapy of prostate cancer (282), and targeting of VISTA on TAMs can be suggested as next therapeutic approach to develop.

Monitoring of serum YKL-40 concentrations can also be considered as promising prognostic approach for the management of CRPC. Thus, post-treatment increase of serum YKL-40 was an independent prognostic factor of earlier death in 106 metastatic prostate cancer patients (Denmark cohort) treated with total androgen ablation or parenteral estrogen (283). Retrospective analysis of 109 patients with CRPC receiving first-line chemotherapy with docetaxel revealed significance of high pre-treatment YKL-40 serum levels as predictive parameter of shorter OS and DSS (284).

These data demonstrate the essential role of TAMs in prostate cancer progression and emphasize on the promise of targeting TAMs to prevent the recurrence of disease and achieve sustained improvements in patient outcomes. Further in-depth investigations must be done to characterize macrophage phenotypes within certain intratumor compartments of prostate cancer and determine their potential diagnostic and therapeutic value.

## Conclusions

In our review we compile existing lines of evidence about the clinical role of TAMs in the context of metastasis (including survival rate) and antitumor treatment in different cohorts of patients that come out of a number of courtiers worldwide. We compared the role of TAMs in worldwide leading types of malignant diseases: breast, colorectal, lung, ovarian, and prostate cancers that very frequently give life-threatening distant metastasis. Systematic analysis of TAM biomarkers identified that CD68, and in some cases CD163, are the best markers for the quantification of TAMs in tumor tissue, while several other surface receptors (scavenger receptor stabilin-1, mannose receptor CD206, CD204, MARCO) and chitinase-like proteins (YKL-39, YKL-40) are very informative biomarkers of functional TAM polarization.

**Figure 2:**
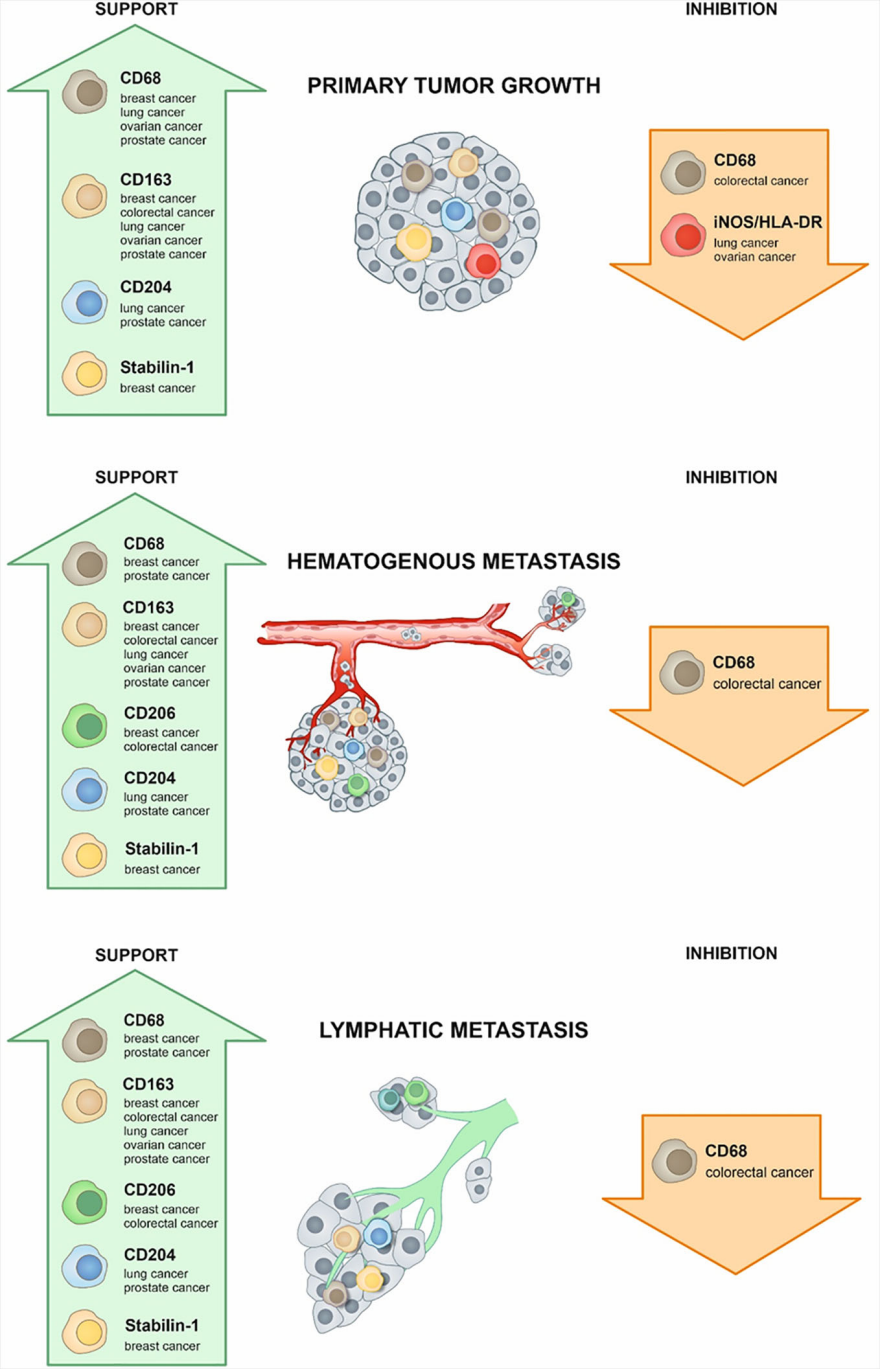
TAMs in primary tumor growth and metastasis. Role of TAMs in primary tumor growth, hematogenous metastasis, and lymphatic metastasis is illustrated. Green arrow indicates supportive role of TAMs for each process, and orange arrow indicates the suppressive role of TAMs. The role of each specific macrophage marker in the individual type of cancer is indicated within the arrows.

In patients with breast, ovarian, and prostate cancer, increased amount of TAMs is a clear indicator for rapid tumor growth, aggressive metastatic process, and limited efficiency of therapy (**Tables 2**–**6**) (**Figure 2**). In lung and ovarian cancer, the major parameter associated with prognosis was not the total amount of CD68+ macrophages, but M1/M2 index. The prevalence of M1 macrophages was favorable for the patients, indicating that in lung tumor M1 TAMs have the ability to limit tumor progression. Moreover, in lung cancer, high amount of TAMs in tumor nest correlated with the chemotherapy efficiency. The most distinct from other types of cancer was colorectal cancer, where high amounts of TAMs were indicative of the favorable prognosis and restricted ability of primary tumors to grow and to metastasize (**Figure 2**). In contrast to the total amount of macrophages, M2-like phenotype of TAMs is rather indicative for the negative prognosis for patients with CRC.

TAMs may contribute to resistance to therapy facilitating tumor progression by suppression of T cell immunity, the maintenance of tumor cell survival, and the stimulation of tumor revascularization. Chemotherapy can stimulate antitumor immunity, thereby increasing the pathological complete response (pCR) to the treatment. There is no agreement about the role of TAMs in chemotherapy response. The results are contradictory and depend on the animal model, type of *in vitro* study, patient cohort, and type of anti-cancer drug (**Table 7**). Therefore, to achieve the maximum efficiency of chemotherapy, the molecular mechanisms of the interaction of chemotherapeutic agents with TAMs have to be investigated. Understanding of these interactions will also allow developing targeting strategies for TAMs. The investigation of TAM-mediated tumor resistance to therapy is of particular relevance in the era of the development of immunomodulatory approaches aimed to enhance T-cell immunity, to inhibit macrophage recruitment into a tumor, to modify polarization of TAMs, and to enhance phagocytosis of cancer cells by TAMs.

**Table 7:** The association of TAMs with the effect of chemotherapy in patients.

Cohort of patient	Method of detection	The type and scheme of chemotherapy (adjuvant, neoadjuvant)	The amount of TAMs in chemotherapy-treated tumors	Correlation of TAM with the effect of chemotherapy	Reference
311 breast cancer patients (Sweden)	Flow cytometry, IHC	Neoadjuvant (PTX and FU-doxorubicin-cyclophosphamide)	fivefold increase of CD45+CD11b+CD14+ macrophage percentage of total cells is found in NAC-treated patients compared to non-treated patients	CD68 high/CD8low ratio is associated with almost fourfold decreased pCR rate compared to cases with CD68low/CD8high ratio (7 *vs*. 27%)	(72)
7 breast cancer patients (USA)	IHC	Neoadjuvant (paclitaxel-based)	Increased amount of CD68+ TAMs in tumor post NAC treatment compared to pre-treatment biopsy	Not studied	(95)
33 breast cancer patients (UK)	IHC	Neoadjuvant (capecitabine plus docetaxel preceded by adriamycin and cyclophosphamide)	Not studied	High CD163+ infiltration (defined as grades 3 and 4) in primary tumor and ALNs are associated with pCR following NAC	(98)
40 breast cancer patients (Russia)	Real-time qPCR	Neoadjuvant (PTX- or taxotere-based)	Not studied	sixfold increase of YKL-39 expression levels after NAC correlates with distant metastasis and poor response to NAC	(17)
123 metastatic CRC patients (Turkey)	IHC	Adjuvant (bevacizumab plus OXP-based or irinotecan-based chemotherapy)	Not studied	Low CD68+ TAM infiltration (scored as <50% staining of stromal cells) is associated with almost twofold longer OS (26.7 ± 8.8 vs. 14.1 ± 1.7 months) and 1.5-fold longer RFS (9.3 ± 1.8 vs. 6.5 ± 1.2 months) after chemotherapy compared to patients with high CD68+ TAM infiltration	(140)
208 stage III CRC patients (Italy)	IHC	Adjuvant (5-FU)	Not studied	Increase of CD68+ TAM immune-reactive area above 8% in primary tumor is associated with increased DFS rate by 30% in 5-FU treated patients with stage III	(141)
521 stage II colon cancer patients (China)	TMA	Adjuvant (FU-based)	Not studied	High CD206+ TAM amount (≥74 cells per ×200 HPF) and increase of CD206/CD68 ratio (above 0.77) correlate with decreased DFS and OS rates after postoperative FU-based therapy by 20% and 30-40%, respectively.	(124)
163 stage II/III NSCLC patients (USA)	Multiplex IF	Neoadjuvant (platinum-based)	twofold increase of CD68+ TAM median density in NAC-treated compared to untreated patients (609.36 *vs*. 298.8 cells/mm^2^)	Increase of epithelial and stromal CD68+ TAM densities above the medians (17 and 25 cells/mm^2^, respectively, under ×200) correlate with increased OS rate by almost 20% in patients who received NCT	(186)
27 stage IIIA NSCLC patients (China)	IHC (manually)	Neoadjuvant (cisplatin/docetaxel)	Not studied	Decrease of CD68+ TAM amount below the median (<222 cells per HPF ×200) is associated with threefold longer DFS (median=16.3 *vs*. 5.3 months in high CD68+ TAMs). High islet/stromal CD68+ TAM ratio (>1.33) correlates with almost fourfold longer DFS (median = 20.7 *vs*. 5.5 months) and longer OS (unreached *vs*. 34.8 months) compared to low ratio	(187)
140 ovarian cancer patients (Italy)	Flow cytometry	Adjuvant (cisplatin/carboplatin + Taxol + bevacizumab)	twofold increase of M1/M2 ratio is found in platinum-sensitive tumors compared to platinum-resistant tumors (2.6 ± 1.1 *vs*. 0.7 ± 0.2).	High M1/M2 ratio (≥1.4) is associated with almost twofold longer OS (34 *vs*. 18 months) and almost threefold longer PFS (24 *vs*. 9 months) compared to those with low M1/M2 ratio	(210)

ALN, axillary lymph node; CRC, colorectal cancer; DFS, disease-free survival; DSS, disease-specific survival; FU, fluorouracile; HPF, high-power field; IF, immunofluorescence; IHC, immunohistochemistry; LN, lymph node; NAC, neoadjuvant chemotherapy; NSCLC, non-small cell lung cancer; OS, overall survival; PFS, progression-free survival; pCR, pathological complete response; RFS, recurrence-free survival; TAMs, tumor-associated macrophages; TMA, tissue microarray.

## Author Contributions

Conceptualization: IL and JK. Writing—original draft preparation: IL, JK, GT, AP, MS, VP, EC, and NC. Writing—review and editing: IL, GT, and JK. Figure preparation: IL. Supervision: JK. Funding acquisition: JK. All authors contributed to the article and approved the submitted version.

## Funding

This research was funded by the Russian Science Foundation, grant number #19-15-00151. The publication costs were covered by the Tomsk State University competitiveness improvement program.

## Conflict of Interest

The authors declare that the research was conducted in the absence of any commercial or financial relationships that could be construed as a potential conflict of interest.

## Abbreviations

ADT, androgen deprivation therapy; BC, breast cancer; CRC, colorectal cancer; CRPC, castration-resistant prostate cancer; DFS, disease-free survival; DSS, disease-specific survival; EMT, epithelial–mesenchymal transition; EOC, epithelial ovarian cancer; IF, immunofluorescence; IHC, immunohistochemical analysis; LN, lymph node; LVI, lymphovascular invasion; NAC, neoadjuvant chemotherapy; NSCLC, non-small cell lung carcinoma; OC, ovarian cancer; OS, overall survival; OXP, oxaliplatin; PC, prostate cancer; pCR, pathologic complete response; PFS, progression-free survival; RFS, relapse-free survival; PTX, paclitaxel:TAMs, tumor-associated macrophages; TMA, tissue microarray; TME, tumor microenvironment; TNBC, triple-negative breast cancer; TN, tumor nest; TS, tumor stroma; PSA, prostate-specific antigen.
